# Blood–brain barrier opening as a predictor of epilepsy and mortality after subarachnoid haemorrhage

**DOI:** 10.1016/j.ebiom.2025.106018

**Published:** 2025-11-11

**Authors:** Jens P. Dreier, Svetlana Lublinsky, Viktor Horst, Sebastian Major, Coline L. Lemale, Vasilis Kola, Maren K.L. Winkler, Eun-Jeung Kang, Karl Schoknecht, Nils Hecht, Anna Maslarova, Edgar Santos, Johannes Platz, Christina M. Kowoll, Oliver W. Sakowitz, Erdem Güresir, Hartmut Vatter, Christian Dohmen, Stefan Wolf, Michael Scheel, Peter Vajkoczy, Jed A. Hartings, Johannes Woitzik, Peter Martus, Alon Friedman

**Affiliations:** a Centre for Stroke Research Berlin, Charité – Universitätsmedizin Berlin, Corporate Member of Freie Universität Berlin, Humboldt-Universität zu Berlin, and Berlin Institute of Health, Berlin, Germany; b Department of Experimental Neurology, Charité – Universitätsmedizin Berlin, Corporate Member of Freie Universität Berlin, Humboldt-Universität zu Berlin, and Berlin Institute of Health, Berlin, Germany; c Department of Neurology, Charité – Universitätsmedizin Berlin, Corporate Member of Freie Universität Berlin, Humboldt-Universität zu Berlin, and Berlin Institute of Health, Berlin, Germany; d Bernstein Centre for Computational Neuroscience Berlin, Berlin, Germany; e Einstein Centre for Neurosciences Berlin, Berlin, Germany; f Departments of Brain and Cognitive Sciences, Physiology and Cell Biology, Faculty of Health Sciences, Ben-Gurion University of the Negev, Soroka University Medical Centre, Beer-Sheva, Israel; g Institute of Neuropathology, Charité – Universitätsmedizin Berlin, Corporate Member of Freie Universität Berlin, Humboldt-Universität zu Berlin, and Berlin Institute of Health, Berlin, Germany; h Berlin Institute of Health at Charité – Universitätsmedizin Berlin, QUEST Centre for Responsible Research, Charitéplatz 1, 10117, Berlin, Germany; i Robert Koch-Institute, Berlin, Germany; j Carl Ludwig Institute for Physiology, Medical Faculty, University of Leipzig, Leipzig, Germany; k Department of Neurosurgery, Charité – Universitätsmedizin Berlin, Corporate Member of Freie Universität Berlin, Humboldt-Universität zu Berlin, and Berlin Institute of Health, Berlin, Germany; l Department of Neurosurgery, University Hospital and Friedrich-Wilhelms-University Bonn, Bonn, Germany; m Neuroscience Institute, New York University Grossman School of Medicine, New York, USA; n Department of Neurosurgery, Heidelberg University Hospital, Ruprecht-Karls-University Heidelberg, Germany; o Department of Neurosurgery, Goethe-University Frankfurt, Germany; p Department of Neurology, University of Cologne, Faculty of Medicine and University Hospital Cologne, Cologne, Germany; q Märkische Kliniken Lüdenscheid, Department of Neurology, Lüdenscheid, Germany; r Department of Neurosurgery, Medical Faculty, University of Leipzig, Leipzig, Germany; s Department for Neurology and Neurological Intensive Care Medicine, LVR-Klinik Bonn, Bonn, Germany; t Department of Neuroradiology, Charité – Universitätsmedizin Berlin, Corporate Member of Freie Universität Berlin, Humboldt-Universität zu Berlin, and Berlin Institute of Health, Berlin, Germany; u Department of Neurosurgery, University of Cincinnati College of Medicine, Cincinnati, OH, USA; v Department of Neurosurgery, Evangelisches Krankenhaus Oldenburg, University of Oldenburg, Oldenburg, Germany; w Institute for Clinical Epidemiology and Applied Biometry, University of Tübingen, Tübingen, Germany; x Departments of Medical Neuroscience, Paediatrics and Surgery, Faculty of Medicine, Dalhousie University, Halifax, Nova Scotia, Canada

**Keywords:** Blood–brain barrier, Epilepsy, Spreading depolarisation, Electrographic seizure, Subarachnoid haemorrhage, Neurodegeneration

## Abstract

**Background:**

We determined the predictive power of semi-automated blood–brain barrier assessment and other variables collected during neurocritical care for the outcome of ‘epilepsy or late death’ following aneurysmal subarachnoid haemorrhage.

**Methods:**

This is a secondary analysis of the prospective, non-interventional, prognostic DISCHARGE-1-cohort from six university hospitals in Germany. All patients who underwent at least one contrast-enhanced MRI during neurocritical care were included. Initial clinical scores and Modified Rankin Scale at day 14 were available. Subdural electrocorticography was scored for seizures and spreading depolarisations. Two MRIs, one post-aneurysm occlusion and another post-neuromonitoring, were semi-automatically segmented into cerebrospinal fluid spaces, normal brain tissue, and abnormal brain tissue. Normal and abnormal tissue were further classified into tissue with “intact” or “dysfunctional” blood–brain barrier. Epilepsy and late death were determined at a median of 3.7 years.

**Findings:**

Abnormal, barrier-dysfunctional tissue as a percentage of intracranial volume on post-monitoring MRI was the only independent predictor of early death within three weeks among 130 patients. In the 121 early survivors, this variable was also the only independent predictor of ‘epilepsy or late death’. This result, obtained by a combination of imputation and the leaving-one-out method, was confirmed in two sensitivity analyses within smaller populations and with fewer missing values.

**Interpretation:**

The study substantiates previous experimental evidence that blood–brain barrier dysfunction plays a key role in epileptogenesis after brain injuries. Contrast-enhanced MRI, a minimally invasive technique, highlighted abnormal, barrier-dysfunctional tissue as a stand-alone independent predictor, underscoring its potential as a ‘precision medicine’ tool in early diagnosis and intervention.

**Funding:**

JPD and AF report a grant from the Era-Net Neuron EBio2 with funds from BMBF 01EW2004 and CIHR Award No. NDD 168164. JPD reports a grant from DFG DR 323/10-2 (project number: 413848220) and EU Horizon MSCA-DN 101119916—SOPRANI. AF reports grants from the 10.13039/501100000024Canadian Institutes of Health Research (CIHR) PJT 148896 and 10.13039/501100003977Israel Science Foundation (ISF) 2254/20. NH is Berlin Institute of Health Clinical Fellow, funded by 10.13039/501100013865Stiftung Charité.

**Research in context**

**Evidence before this study**

We searched PubMed for articles published in any language addressing the possible association between blood–brain barrier (BBB) damage in patients with subarachnoid haemorrhage (SAH) or in animals subjected to experimental SAH and the risk of developing late-onset epilepsy or death. We used the search terms “blood–brain barrier”, “BBB”, “magnetic resonance imaging”, “MRI”, “subarachnoid h(a)emorrhage”, “SAH”, “epilepsy”, and “mortality”. Our last search was conducted on May 28, 2025. We found no reports that investigated the possible association between BBB dysfunction during the acute or subacute phase of SAH and the development of epilepsy or death in patients or animals after SAH. Under the search terms “blood–brain barrier”, “BBB”, “stroke” and “epilepsy”, we found a review from 2024 which, building on the experimental findings on post-traumatic epilepsy, examined whether BBB dysfunction is a potential contributor to epileptogenesis after ischaemic or haemorrhagic stroke. Meijer and Gorter explicitly addressed the level of evidence for this hypothetical association in their abstract: “Nevertheless, studies directly comparing BBB dysfunction and poststroke epilepsy are largely absent”. Overall, the predictive power of BBB dysfunction for epileptogenesis in clinical settings, including traumatic brain injury and stroke, remains to be established.

**Added value of this study**

Our study is the first to fill this gap. It used a large multicentre cohort of 130 patients with aneurysmal SAH, of whom 279 contrast-enhanced MR images of the brain were analysed, to test the potential of quantitative BBB imaging as a prognostic biomarker for the development of post-stroke epilepsy and mortality. Four semi-automatically segmented predictive variables were examined for each MR scan performed on median day 2 and day 15 after the initial haemorrhage. Invasive neuromonitoring in the form of subdural electrocorticography during the first two weeks was scored for epileptic seizures and spreading depolarisations. Other predictive variables examined were initial clinical scores such as the World Federation of Neurosurgical Societies Score and the Rosen Macdonald Score as well as the value of the post-monitoring Modified Rankin Scale. Epilepsy and late death were determined at a median of 3.7 years. Abnormal, barrier-dysfunctional tissue on post-monitoring MRI was the only independent predictor of early death within three weeks among 130 patients. In the 121 three-week survivors, the same variable emerged as the sole predictor of the binary outcome ‘post-stroke epilepsy or late death’ after applying Bonferroni correction. This was also the only variable left in multivariate analysis employing forward selection. Two sensitivity analyses confirmed the result.

**Implications of all the available evidence**

Since 2004, an increasing number of experimental studies have suggested that BBB dysfunction is involved in the initiation of transcriptional changes in the neurovascular network that ultimately lead to epileptogenesis and neurodegeneration after traumatic brain injury. Clinical correlation studies found that patients with post-traumatic epilepsy often show chronic BBB dysfunction. This hypothesis has been discussed in several top-level reviews by different groups and has recently been extended to epileptogenesis after stroke. What has been missing so far, and what our study provides for the first time using semi-automated MRI segmentation, is evidence from a clinical study that BBB dysfunction in the early phase after cerebral injury is predictive of late epileptogenesis and mortality. Semi-automated MRI segmentation thus emerges as a potential powerful tool for ‘individualised medicine’ to guide anti-epileptogenic and anti-neurodegenerative interventions after cerebral injuries.

## Introduction

Post-stroke epilepsy (PSE) is a major complication of stroke, and is associated with increased mortality and morbidity.^1^, ^2^, ^3^ In the United States, an estimated 11% of all epilepsies are due to stroke,^4^ with incidences notably higher following haemorrhagic stroke.^5^^,^^6^ For instance, a study of patients aged ≥60 years identified haemorrhagic stroke as having the strongest association with epilepsy (odds ratio (OR) = 3.31), followed by ischaemic stroke (OR = 2.32) and alcohol-related disorders (OR = 2.20).^7^ Since 2004, an increasing number of experimental and clinical correlation studies have suggested that blood–brain barrier dysfunction (BBBD) could be involved in post-traumatic epilepsy (PTE), via transforming growth factor-β (TGFβ) receptor-activated signalling in astrocytes.^8^, ^9^, ^10^, ^11^, ^12^, ^13^, ^14^, ^15^, ^16^, ^17^, ^18^ On this basis, the additional hypothesis was formulated that BBBD could also be involved in the development of PSE, although experimental or clinical studies directly comparing BBBD and PSE are largely absent.^19^

Subarachnoid haemorrhage (SAH), the second most common type of haemorrhagic stroke, shows a global incidence of 6.1 (95% confidence interval (95% CI): 4.9–7.5) per 100,000 person years^20^ and accounts for ∼5% of all strokes.^21^ The median case fatality rate varies between 27 and 44% across different regions,^22^^,^^23^ with a median age of occurrence being 55 years and a median age at death of 59 years.^24^, ^25^, ^26^ Because of the high mortality and young age, it is estimated that 27.3% of all potential life years lost to stroke before age 65 (YPLL)–a measure of premature mortality–are attributable to SAH.^26^ SAH is caused by a ruptured aneurysm in 85% of cases. Women suffer from aneurysmal subarachnoid haemorrhage (aSAH) more often than men.^21^ In Germany, the female to male ratio is 1.8:1.0.^27^

Aside from causing severe motor, cognitive and emotional disabilities, PSE is a dreaded long-term complication of aSAH.^28^^,^^29^ Patients who develop PSE show no functional recovery on the Modified Rankin Scale (MRS) between three and twelve months after aSAH.^30^ Moreover, PSE is associated with a higher risk of mortality during long-term follow-up among twelve-month survivors of aSAH.^31^

To reduce this burden, there is an urgent need to improve treatment by discovering early mechanistic biomarkers that predict PSE and poor outcome and guide targeted interventions. This is the first study to evaluate the predictive power of BBBD for ‘PSE or late death’ in a clinical cohort. BBBD was semi-automatically quantified using gadolinium diethylene-triamine-pentaacetic acid (Gd-DTPA)-enhanced, i.e., contrast-enhanced (CE)-MRIs. Additional predictive variables examined were (i) initial clinical scores,^32^, ^33^, ^34^ (ii) early seizures,^28^^,^^29^ and (iii) spreading depolarisations (SD),^35^^,^^36^ continuously recorded by subdural electrocorticography (ECoG) during the first 15 days, and (iv) post-neuromonitoring MRS.

## Methods

### Patients

**Fig. 1:**
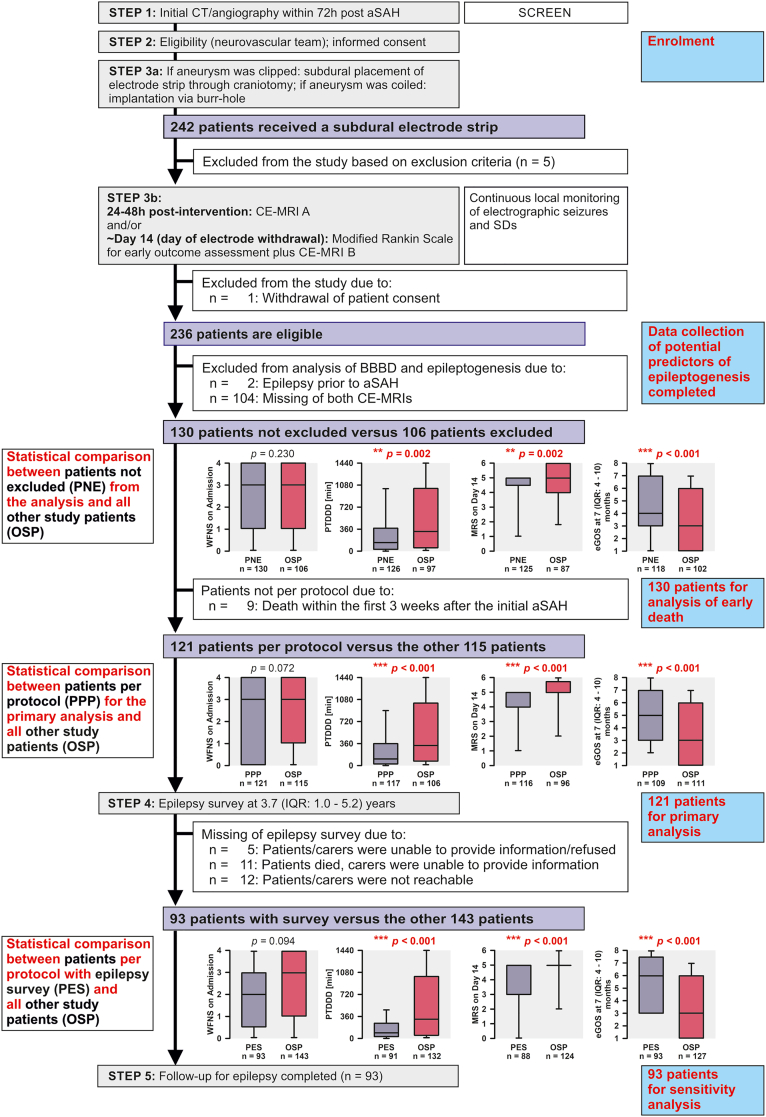
**Patient flow and entry into analysis**. All 242 patients in the prospectively collected database were recruited either as part of the bi-centre pilot study in preparation for the ‘Depolarisations in ISCHaemia after subARachnoid haemorrhaGe-1’ (DISCHARGE-1) trial between April 2005 and March 2009 (n = 37) or as part of the multicentre DISCHARGE-1 trial between September 2009 and April 2018 (n = 205). Out of 242 patients, six were excluded because they did not meet the inclusion/exclusion criteria or their informed consent was withdrawn, leaving 236 patients. Our main interest in the present study was to determine whether there is an association between BBBD in the first two weeks after the initial aSAH and the combined endpoint ‘development of post-stroke epilepsy (PSE) or late death’. Therefore, we excluded two patients who already had epilepsy before aSAH and 104 patients who had neither undergone early CE-MRI_early_ nor CE-MRI_post-monitoring_, precluding assessment of BBBD. Thus, the first target population of our analysis consists of the remaining 130 patients, i.e., the population of patients without previous epilepsy and with at least one available CE-MRI obtained during neurocritical care. In the interest of patient safety, CE-MRI had to be optional, but this led to a selection bias, as sicker patients were more likely to be excluded. Thus, the statistical analyses shown in the figure demonstrate that two of the early indicators of poor long-term outcome, namely PTDDD and MRS_Day14_,^24^ as well as the eGOS score, were significantly worse in the patients who were excluded than in those who remained in the analysis. Of the 130 patients who were not excluded, nine patients died in hospital within three weeks of the initial aSAH, so by definition they had no chance of developing epilepsy. In this population, we investigated whether variables collected during neurocritical care predicted early death within 21 days. In contrast, the aim of the primary analysis was to predict the combined endpoint ‘development of PSE or late death’ in patients who survived the first 21 days. We therefore conducted the primary analysis in the 121 patients who belonged to the first target population and additionally survived at least 21 days. Of the 121 patients, 28 patients had missing information on epilepsy (16 with successful contact of whom 11 had died without information on possible preceding epileptic seizures, and 12 patients without contact). For the remaining 93 patients, only missing data on the predictors had to be imputed. We chose this population for a sensitivity analysis because no meaningful target population could be defined at the time of the prognostic measurements. The statistical comparisons shown in the figure were performed using Mann–Whitney Rank Sum Tests. aSAH, aneurysmal subarachnoid haemorrhage; BBBD, blood–brain barrier dysfunction; CE-MRI, contrast-enhanced magnetic resonance imaging; CT, computed tomography; eGOS, extended Glasgow outcome Scale; MRS, Modified Rankin Scale; OSP, other study patients; PES, patients per protocol with epilepsy survey; PNE, patients not excluded; PPP, patients per protocol; PTDDD, peak total spreading depolarisation-induced depression duration of a recording day; SD, spreading depolarisation; WFNS, World Federation of Neurosurgical Societies score.

We selected patients from a prospective database based on predefined criteria as described in Fig. 1. All patients were recruited either as part of the bi-centre pilot study in preparation for the ‘Depolarisations in ISCHaemia after subARachnoid haemorrhaGe-1’ (DISCHARGE-1) trial between April 2005 and March 2009 (n = 37; Campus Benjamin Franklin and Campus Virchow Klinikum, Charité-Universitätsmedizin Berlin) or as part of the multicentre DISCHARGE-1 trial between September 2009 and April 2018 (n = 199).^24^^,^^37^ Both studies shared identical inclusion and exclusion criteria in addition to relevant aspects of patient management such as sedative and anticonvulsant regimens.

**Fig. 2:**
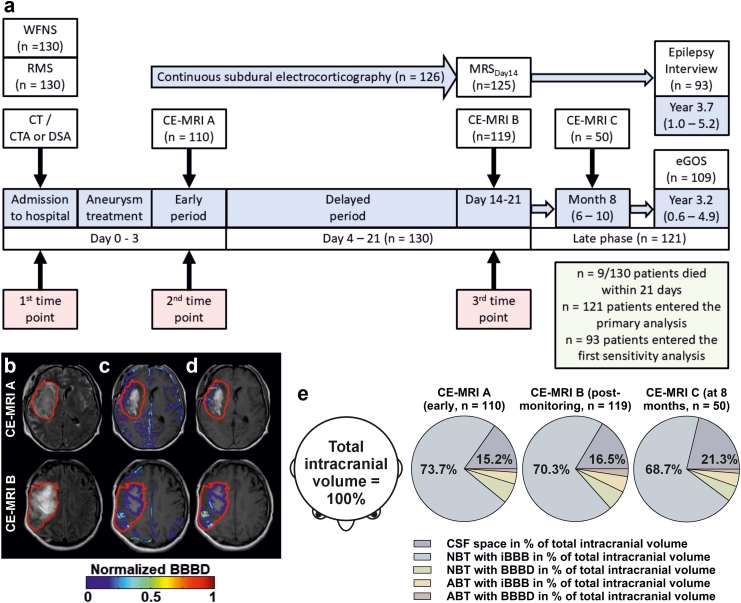
**Study flow**. (a) In every patient, the first 24-h period after the initial haemorrhage was always denoted as day 0, the second 24-h period as day 1 and so on.^24^^,^^38^ Aim of our primary statistical analysis was to define prognostic factors for the combined endpoint ‘post-stroke epilepsy (PSE) or late death’. Variables were collected at three time points during neurocritical care (1st, 2nd and 3rd time point). CT and CTA were performed on admission. If necessary, CTA was complemented by DSA. BBBD, blood–brain barrier dysfunction; CE-MRI, contrast-enhanced magnetic resonance imaging; CE-MRI A, CE-MRI_early_ on median day 2 (interquartile range (IQR): 1–3); CE-MRI B, CE-MRI_post-monitoring_ on median day 15 (IQR: 13–16); CE-MRI C, CE-MRI_late_ on median month 8 (IQR: 6–10); CT, computed tomography; CTA, computed tomography angiography; DSA, digital subtraction angiography; eGOS, extended Glasgow Outcome Scale; MRS, Modified Rankin Scale; RMS, Rosen Macdonald Score^39^; WFNS, World Federation of Neurosurgical Societies score.^40^ (b) The detected abnormal brain tissue (ABT) quantified by the semi-automated approach^41^ is shown within the red border in the Fluid Attenuated Inversion Recovery (FLAIR) image. Accordingly, the normal brain tissue (NBT) is shown outside of the red border. In CE-MRI A (day 2), the ABT resulted from a right-sided Sylvian haematoma with intracerebral extension and perifocal oedema in this example. In CE-MRI B (day 16), the increase of ABT was due to a delayed cerebral infarct. (c) and (d) Enhancement maps of BBBD, superimposed on the same T1-weighted MRI. (c) Shows BBBD in the whole brain slice and (d) shows ABT_BBBD_ within the red border. BBBD enhancement maps were created by reassigning each voxel with BBBD its enhancement level.^41^ The final BBBD enhancement map was normalised to a range of 0–1 (minimum to maximum enhancement value, compare colour code below the figure). The detected ABT is again shown within the red border. (e) The figure explains the five semi-automatically segmented compartments in CE-MRI A, CE-MRI B and CE-MRI C. The sizes of the five compartments shown correspond to the mean values of these compartments as a percentage of the total intracranial volume in the respective MRI. In the body text, the following abbreviations are used for the five compartments: ABT_BBBD_%, ABT with BBBD in % of total intracranial volume; ABT_iBBB_%, ABT with intact BBB in % of total intracranial volume; CSF space, internal and external cerebrospinal fluid space; NBT_BBBD_%, NBT with BBBD in % of total intracranial volume; NBT_iBBB_%, NBT with intact BBB in % of total intracranial volume.

The primary objective of the present study, which is a secondary analysis of the DISCHARGE-1 trial data, was to assess the predictive power of neurocritical care variables for the binary outcome of ‘PSE or late death’ after aSAH. This was restricted to all patients who had at least one CE-MRI to assess the BBB during neurocritical care, and who survived at least 21 days. Evaluators were blinded to other measures. Fig. 2a shows the study flow. Further objective was a similar approach for the outcome death until day 21 including nine more patients.

### Inclusion and exclusion criteria

Inclusion criteria were: (i) age (≥18 years); (ii) World Federation of Neurosurgical Societies (WFNS) grade I–V; (iii) ruptured saccular aneurysm proven by CT-angiography or digital subtraction angiography (DSA); (iv) symptom onset within the preceding 72 h; (v) either surgical treatment of the aneurysm via craniotomy or, in coiled patients, burr hole trepanation for placement of an external ventricular drain or oxygen sensor, which allows the simultaneous placement of a subdural electrode strip (Wyler, platinum-iridium, 6-contact, 10-mm electrode spacing, 5-mm diameter, Ad-Tech Medical, Racine, Wisconsin, USA); and (vi) informed consent is obtained from the patient or a legal representative.^24^^,^^37^

Exclusion criteria were: (i) SAH due to other causes (e.g., trauma, fusiform or mycotic aneurysm); (ii) admission in a clinical state with unfavourable prognosis (e.g., wide, nonreactive pupils for more than 1 h), (iii) bleeding diathesis (thrombocytes <60/nl, Quick value <60%, partial thromboplastin time >45s); (iv) cytostatic therapy in patients with malignant disease; (v) pregnancy; (vi) unavailability of the monitoring equipment or sufficient staff; and (vii) refusal of the patient or legal representative to participate in the study.^24^^,^^37^

Exclusion criteria for performing MRI that required a CT to be performed instead of an MRI scan were: (i) non-MRI-compatible, non-removable metals, artificial joints etc., or electronic devices (pacemaker, pumps etc.); and (ii) respiratory and/or haemodynamic instability not allowing MRI transport. Since the neuroimaging protocol including CE-MRI required more time, this examination was only carried out in selected patients who were classified as sufficiently stable by the accompanying intensivist at the time of the examination. In addition, allergies and kidney failure were specific exclusion criteria for CE-MRI.

In contrast to the DISCHARGE-1 trial,^24^ the following two criteria did not lead to patient exclusion: (i) ECoG recording time of less than 24 h between the neuroimage for the assessment of the tissue loss due to early focal brain injury and the neuroimage for the assessment of the tissue loss due to delayed cerebral ischaemia (DCI); or (ii) a missing post-monitoring image in early survivors.

### Patient management

As described, patients were managed according to local standard care, including oral nimodipine prophylaxis, daily transcranial Doppler-sonography (TCD) and a DSA on day 7 (interquartile range (IQR): 7–8) to determine the degree of angiographic vasospasm.^24^ First-line sedative was propofol. Midazolam was the preferred agent for sedation in patients requiring prolonged ventilation. Analgesia was provided with fentanyl, its analogues, and morphine. No other anticonvulsants than midazolam and propofol were given for seizure prophylaxis. Patients developing seizures during neurocritical care were treated with levetiracetam.

At the conclusion of surgery, a linear ECoG strip was placed on the cerebral cortex, targeting the vascular territory of the aneurysm-bearing vessel. After surgery, patients were transferred to neurocritical care and continuous neuromonitoring was initiated. Arterial pressure was recorded from the radial artery. Intracranial pressure was monitored using an external ventricular drain or an intracranial pressure transducer (Codman or Camino systems). Glasgow Coma Scale was documented every 4 h. Blood gases, glucose and electrolytes were documented every 6 h. A thorough neurological examination was performed at least daily. ECoG was terminated when invasive neuromonitoring was no longer clinically required or by day 14 post haemorrhage. At the conclusion of the monitoring period, the subdural electrode strip was removed at the bedside by gentle traction.

### Ethics

The protocol was approved by the ethics committees of Charité—Universitätsmedizin Berlin, corporate member of Freie Universität Berlin, Humboldt-Universität zu Berlin, and Berlin Institute of Health, Berlin, Germany (EA4/070/23), University Hospital and Friedrich-Wilhelms-University Bonn, Bonn, Germany (Lfd. Nr. 013/15), University of Cologne, Faculty of Medicine and University Hospital Cologne, Cologne, Germany (Geschäftsnr. 10–100), Goethe-University Frankfurt, Frankfurt, Germany (Geschäftsnr. 40/10), Heidelberg University Hospital, Ruprecht-Karls-University Heidelberg, Germany (S-456/2009).^24^^,^^37^ Either informed consent or surrogate informed consent was obtained. Research was conducted in accordance with the Declaration of Helsinki. DISCHARGE-1 was preregistered (http://www.isrctn.com/ISRCTN05667702). Results are reported following the STROBE guidelines (https://www.strobe-statement.org).

### MRI acquisition

As the patients were enrolled in different centres during more than a decade, image acquisition slightly varied in scanner type, protocol and sequence parameters (Table 1). MRI was performed on 1.5T or 3T scanners. The protocol typically included axial T2-weighted (T2w) fluid attenuation inversion recovery (FLAIR) images, an axial T2∗w gradient echo sequence, axial multi-shot echo-planar diffusion-weighted imaging (DWI) and corresponding apparent diffusion coefficient (ADC) maps, spin echo T1-weighted (T1w) images before and 5 min after the injection of Gd-DTPA (0.5 mol/L, 0.1 mmol/kg body weight), and a 3D T1w magnetisation prepared rapid gradient echo (MPRAGE) sequence with isotropic 1 mm^3^ voxels. The slice thickness varied between 5 and 6 mm.

**Table 1:** MRI multi-centre standard sequences description.

Study centre	Patients	Scanner	Parameters	T1w	DWI	FLAIR
Campus Benjamin Franklin, Berlin, Germany	n = 53	Magnetom Avanto Siemens 1.5T	TR/TE (ms) TI (ms) b-value (s/mm^2^) Voxel size (mm^3^) Flip angle (°)	500/7.8 – – 0.45 × 0.45 × 6 88	2900/88 – 1000 1.8 × 1.8 × 6 90	9000/113 2500 – 0.49 × 0.49 × 6 180
Campus Virchow Klinikum, Berlin, Germany	n = 57	Philips Medical Systems Intera 1.5T	TR/TE (ms) TI (ms) b-value (s/mm^2^) Voxel size (mm^3^) Flip angle (°)	565/14 – – 0.45 × 0.45 × 6 90	4347/95 – 1000 0.9 × 0.9 × 6 90	6000/150 2000 – 0.45 × 0.45 × 6 90
Bonn, Germany	n = 8	GE Discovery MR750w 3T	TR/TE (ms) TI (ms) b-value (s/mm^2^) Voxel size (mm^3^) Flip angle (°)	820/13 – – 0.47 × 0.47 × 5 58	6554/77.8 – 500/1000 0.94 × 0.94 × 4 90	10,000/103 2700 – 0.47 × 0.47 × 5 160
Heidelberg, Germany	n = 7	Magnetom Espree eco Siemens 1.5T	TR/TE (ms) TI (ms) b-value (s/mm^2^) Voxel size (mm^3^) Flip angle (°)	500/12 – – 0.45 × 0.45 × 6 90	3600/99 – 1000 1.9 × 1.9 × 6 90	10,000/133 2400 – 0.9 × 0.9 × 6 180
Cologne, Germany	n = 5	Philips Medical Systems Intera 1.5T	TR/TE (ms) TI (ms) b-value (s/mm^2^) Voxel size (mm^3^) Flip angle (°)	450/15 – – 0.9 × 0.9 × 6 90	4446/95 – 1000 0.9 × 0.9 × 6 90	6000/100 2000 – 0.9 × 0.9 × 6 90

DWI, Diffusion Weighted Imaging; FLAIR, Fluid-Attenuation Inversion Recovery MRI; T1w, T1-weighted MRI; TE, Time to Echo; TI, Time to Inversion; TR, Time to Repetition. The characteristics of the scanners mainly used in the centres are shown.

### Multimodal abnormal brain tissue (ABT) segmentation

We have previously developed a method for brain tissue segmentation in MATLAB (MathWorks, version 2017b, USA) based on three steps: (1) preprocessing, (2) initialisation, and (3) segmentation.^41^ The multimodal pipeline uses T1w, FLAIR, and DWI images.

#### Step 1: pre-processing

Pre-processing was performed with SPM12. All MR scans were bias-corrected for MR field inhomogeneity, co-registered to the T1w image using linear (12-parameter affine) and nonlinear transformations and normalised to Montreal Neurological Institute (MNI) space as follows:

1. DWI and FLAIR images were first co-registered to the T1w using SPM12's Coregister function (rigid-body transformation).
2. The T1w was then normalised to MNI space using SPM12's Normalise function, which applies an affine transformation followed by nonlinear warping.
3. The resulting deformation fields were applied to the co-registered DWI and FLAIR images.

The normalised, bias-corrected T1w was used for tissue classification into grey matter (GM), white matter (WM), and cerebrospinal fluid space (CSF), based on Statistical Parametric Mapping (SPM) tissue probability maps. The skull peeled mask was created as a combination of GM, WM, and CSF. The mask was required for anatomical localisation of voxels in the skull peeled volume of interest.

#### Step 2: initialisation

In the images, the initial SPM-based segmentation contained overlap between ABT and GM, WM, and CSF. In order to estimate the potential ABT class, an outlier detection technique was used. At its core was the comparison of patients’ MR images to specially created templates based on healthy control data (using imaging of 21 healthy control subjects, 28.1 ± 14.7 years (range: 19–46 years)).

The T1w, DWI and FLAIR reference templates were developed by averaging corresponding scans of healthy subjects within the skull-peeled volume of interest. The MR intensities corresponding to the pathological structures do not conform to the intensity distribution of the normal structures. Thus, the intensities of pathological structures such as *ABT* represent outliers in the normal intensity distribution defined by reference templates.

Due to differences in multicentre MR scanners and image acquisition protocols and the heterogeneity of healthy and pathological anatomy, MR data and templates’ transformation of the data into z-score space was performed:

${Zscore}_{m}^{s}=\left(I_{m}^{s}-\mu_{m}^{s}\right)/\sigma_{m}^{s}$, where μ and σ are mean and standard deviation of an image I within SP-VOI; *s = {template, patient}* and *m = {T1w, DWI, FLAIR}*. Since the upper tail of FLAIR intensities’ distribution could be influenced by the amount of hyperintense *ABT* voxels, $\mu_{FLAIR}^{patient}$ and $\sigma_{FLAIR}^{patient}$ were trimmed by cutting off the top λ% of voxels. λ was empirically set to 3% with the possibility of manual adjustment, depending on *ABT* size.

Similarity of the templates and corresponding patient's MR data transformed into z-score space was evaluated by means of the Mahalanobis distance:

$$d_{m}^{mahal}=\sqrt{{\left({Zscore}_{m}^{patient}-\bar{{Zscore}_{m}^{template}}\right)}^{T}{\left(\Sigma_{template}^{m}\right)}^{-1}\left({Zscore}_{m}^{patient}-\bar{{Zscore}_{m}^{template}}\right)}$$

where $\bar{{Zscore}_{m}^{template}}$ and $\Sigma_{template}^{m}$ are mean and covariance of the reference template *m*.

Thus, the initial *ABT* was estimated as an aggregation of T1w, DWI and FLAIR outliers:

$$Initial_{lesion}=\sum_{m=1}^{3}\left(d_{m}^{mahal}\geq 3\right)$$

To further partition these *ABT* regions, we applied k-means clustering (k = 2) on voxel intensities from the DWI channel. This procedure yielded two distinct classes: hyperintense voxels and iso-/hypointense voxels.

Hence, initial labelling consisted of five tissue classes (K = 5): GM, WM, CSF, and two *ABT* classes.

#### Step 3: segmentation

The goal of segmentation was to assign each voxel *i* to one of *K* tissue classes based on initial labelling and MR dataset. Multimodal MR scans were modelled as multi-dimensional Gaussian distributions. This enabled combining multiple sequences in a single segmentation task. The segmentation problem was solved using expectation-maximisation for Gaussian mixture model.^42^ The input multimodal MR dataset *X* for a patient consisted of *M* vectorised MR sequences as follows:

$$X=\left[\begin{array}{ccc} X_{11} & \cdots & X_{1M}\\ \vdots & \ddots & \vdots \\ X_{N1} & \cdots & X_{NM} \end{array}\right],X\in SP-\text{VOI,}$$

so that $X_{i}=\left\{\begin{array}{ccc} X_{i1}\text{,} & \ldots \text{,} & X_{iM} \end{array}\right\}$, where *N* is the number of voxels included in SP-VOI.

Since the matrix *X* incorporated data from different modalities, the normalisation procedure was essential. As an initial sub-step of the normalisation, z-score transformation was applied to each individual *X* column. For the next sub-step, principal component analysis was devoted to the z-score transformed *X* matrix and, as a result, a new $X_{score}$ data matrix was created. Percent variation explained by each component of $X_{score}$ was calculated based on eigenvalues. Components that accounted for at least 5% of the total variance were retained for further analysis. As a consequence of principal component analysis, the data were presented along maximum variance axes. Other benefits of this procedure were noise reduction and possible computation simplification due to projecting of *X* matrix into lower dimensional space.

The tissue class labelling matrix was denoted by

$$Z=\left[\begin{array}{ccc} Z_{11} & \cdots & Z_{1K}\\ \vdots & \ddots & \vdots \\ Z_{N1} & \cdots & Z_{NK} \end{array}\right]$$

where $z_{ik}$ is a binary class membership component:

$$Z_{ik}=\left\{ \begin{array}{cc} 1 & \text{if}\;\text{voxel}\;i\;\text{belongs}\;\text{to}\;\text{class}\;k\\ 0 & \text{otherwise} \end{array}\right.$$

withk=1,…,K,andi=1,…,N.

The tissue class labelling matrix was denoted by

$$Z=\left[\begin{array}{ccc} Z_{11} & \cdots & Z_{1K}\\ \vdots & \ddots & \vdots \\ Z_{N1} & \cdots & Z_{NK} \end{array}\right]$$

where $z_{ik}$ is binary class membership component:

$$Z_{ik}=\left\{ \begin{array}{cc} 1 & \text{if}\;\text{voxel}\;i\;\text{belongs}\;\text{to}\;\text{class}\;k\\ 0 & \text{otherwise} \end{array}\right.$$

withk=1,…,K,andi=1,…,N.

The mean vector and covariance matrix within a class are described using $\mu_{k}=\left\{\begin{array}{c} \mu_{k1}\text{,}\ldots \text{,}\mu_{kM} \end{array}\right\}$ and $\Sigma_{k}=\left\{\begin{array}{c} \Sigma_{k1}\text{,}\ldots \text{,}\Sigma_{kM} \end{array}\right\}$. The Gaussian mixture model distribution is expressed as a linear superposition of Gaussians in the form of

$$p\left(x\right)=\sum_{k=1}^{K}\omega_{k}N\left(x|\mu_{k},\Sigma_{k}\right)$$

where $N\left(x|\mu_{k},\Sigma_{k}\right)$ denotes the probability density function of the *k*-th Gaussian component with unknown parameters, including mean vector $\mu_{k}$, covariance matrix $\Sigma_{k}$, and mixture proportion (or prior probability) $\omega_{k}$. The $\omega_{k}$ was calculated as the number of voxels in each class divided by *N*. For convenience, we used $\theta =\left\{\mu_{k},\Sigma_{k},\omega_{k}\right\}$ to represent the unknown parameters. The maximum likelihood function was used to estimate set *θ* of the unknown parameters:

$$L\left(\theta \right)=\sum_{i=1}^{N}\log\left[\sum_{k=1}^{K}\omega_{k}N\left(x_{i}|\mu_{k},\Sigma_{k}\right)\right],\text{and}$$

N(xi|μk,Σk)=1(2π)M2|Σk|12exp[−12(xi−μk)TΣk−1(xi−μk)]

EM algorithm was used to iteratively estimate optimal set of θ. First, initial labelling (K = 5), obtained in Step 2 (initialisation), was applied to calculate $\theta^{\left(t=1\right)}$ (t denotes iteration number).

Second (expectation step), the posterior probability of the voxel i was calculated:

$$\gamma_{ik}^{t}=\frac{\omega_{k}^{t}N\left(x_{i}|\mu_{k},\Sigma_{k}\right)}{\sum_{j=1}^{K}\omega_{j}^{t}N\left(x_{i}|\mu_{j},\Sigma_{j}\right)}\;\text{.}$$

Third (maximisation step), $\gamma_{ik}$ was used to update set of θ:

$$\mu_{k}^{t=t+1}=\frac{1}{N_{k}}\sum_{i=1}^{N}\gamma_{ik}^{t}x_{i}{,\text{where}\;N}_{k}=\sum_{i=1}^{N}\gamma_{ik}^{t}$$

$$\Sigma_{k}^{t=t+1}=\frac{1}{N_{k}}\sum_{i=1}^{N}\gamma_{ik}^{t}\left(x_{i}-\mu_{k}^{t}\right){\left(x_{i}-\mu_{k}^{t}\right)}^{T}$$

$$\omega_{k}^{t=t+1}=\frac{N_{k}}{N}\text{.}$$

Finally, expectation step and maximisation step were repeated until convergence of θ.

The $X_{i}\in X$ was classified based on the maximum posterior probability as

$$Z_{ik}=\arg\max_{k=1,\ldots ,K}\gamma_{ik}$$

The *ABT* object was defined as a combination of two tissue classes: $Z_{ABT}=\left\{k\in \left[4,5\right]\right\}\text{.}$

In order to incorporate the information of neighbouring to *ABT* pixels, a refining procedure was applied, which included two sub-steps: region-growing and morphological. First, adjacent to $Z_{ABT}$ pixels *i* (8-connected *ABT* neighbourhood $W_{ABT}$) were examined and determined, based on inclusion criteria, whether they should be included in the *ABT*. Thus, the neighbouring pixels of the growing *ABT* were iteratively merged according to inclusion criteria:

$$i\in Z_{ABT\;}:\left\{\;\left(\max_{k=1,\ldots ,K}\gamma_{ik}-\gamma_{i4}\right)<0.1\vee \left(\max_{k=1,\ldots ,K}\gamma_{ik}-\gamma_{i5}\right)<0.1\vee \left(\gamma_{i4}+\gamma_{i5}\right)\geq 0.5\right\}\;\forall \;\left\{k\in \left[1,2,3\right]\wedge i\in W_{ABT}\right\}$$

Next, morphological refining of the *ABT* grown object was performed. To this end, small disconnected components with volume <3 mm^3^ were detected and removed. Then, slice-wise morphological closing with a distance of 2 pixels was applied. In addition, the small regions (area <16 mm^2^) completely surrounded by *ABT* were slice-wisely included into the final *ABT* object. The final ABT object was subdivided again into hyperintense versus iso- or normointense DWI objects, as described in Step 2 (initialisation).

In summary, MRI scans were corrected for distortions and aligned to a standard brain template during pre-processing. Tissue types (GM, WM, CSF) were classified using SPM12 software. During initialisation, patients' MRI data were compared to templates made from healthy controls to detect ABT. Abnormal regions were identified as statistical outliers. These regions were further classified based on DWI features into two types: (1) hyperintense and (2) iso- or hypointense DWI. During segmentation, a statistical model was applied to assign each brain voxel to a tissue class by combining information from multiple MRI sequences. Data were normalised and reduced in complexity using principal component analysis. A machine learning approach (expectation-maximisation algorithm) refined the tissue classification. Small irrelevant regions were removed, and neighbouring abnormal tissues were incorporated to form the final ABT map.

The method allowed us to semi-automatically segment the MRI scans into lateral ventricles, subarachnoid space, apparently normal brain tissue (NBT) in the combined grey and white matter, or ABT. As previously explained, ABT reflects cytotoxic or vasogenic oedema, gliosis or haemorrhage.^24^

Since there is no gold standard for ABT segmentation, Lublinksy et al. compared this automated method with manual segmentation by an experienced neuroradiologist in a subset of 9 patients for validation.^41^ Both methods showed high similarity, especially when combining information from different MRI sequences (Dice similarity coefficient: 94.6 ± 12.4%). In DISCHARGE-1, two independent evaluators segmented ABT on MRI_post-neuromonitoirng_ semiautomatically (S.L.) and tissue loss due to intracerebral haemorrhage (ICH), early cerebral ischaemia (ECI), and DCI manually (V.H.). Although the methodological approaches for determining the lesion volumes differed fundamentally, both variables correlated strongly (Spearman's ρ: 0.81, *p* < 0.0001, n = 143).^24^

### BBB permeability evaluation

A previously developed method was used to assess BBB permeability using CE-MRI.^43^ Areas where contrast leaked into brain tissue (indicating BBB opening) were detected by comparing signal changes before and after Gd-DTPA injection. For each subject, Gaussian fits to enhancement distribution histograms were estimated for three regions representing (i) tissue that is enhanced after Gd-DTPA injection (temporal muscle), (ii) non-enhanced tissue (mid-vitreous body) and (iii) tracer-rich blood vessels (confluence point between superior sagittal sinus and transverse sinuses). A mid-vitreous body region of interest was selected to exclude noise and a tracer-rich region of blood vessels was selected to exclude normally enhanced vasculature. Two intersection points were used to estimate an enhancement range indicative of brain tissue lacking a normal BBB: intersection between Gaussian fits to mid-vitreous body and muscle (τ_1_) and intersection between Gaussian fits to muscle and blood vessel region (τ_2_). A morphological filtering procedure, based on connected components labelling, was applied such that clusters smaller than four neighbouring voxels were removed. The detected brain tissue with BBBD was used to define the final brain region with BBBD by appending connected neighbouring voxels (8-connectivity) with expanded enhancement range of 0.5τ_1_–1.5τ_2_. A BBBD enhancement map was created by reassigning each voxel of BBBD with its enhancement level. The final BBBD enhancement map was normalised to 0–1 range.

NBT was quantified as a percentage of total intracranial volume (NBT%) and categorised into NBT associated with BBBD (NBT_BBBD_%) and NBT with intact BBB (NBT_iBBB_%) (Fig. 2b–e). Likewise, ABT was also quantified as a percentage of total intracranial volume (ABT%) and divided into ABT associated with BBBD (ABT_BBBD_%) and ABT with intact BBB (ABT_iBBB_%).

### ABT vicinity analysis

**Fig. 3:**
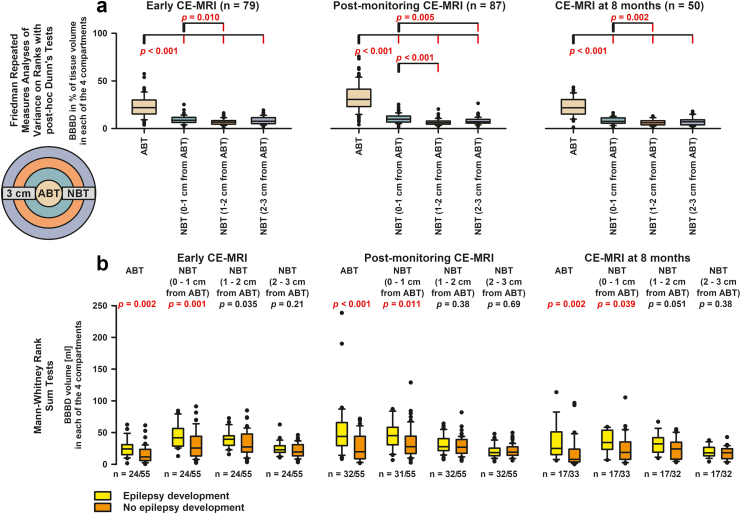
**Spatial analysis**. (a) The proportion of semi-automatically determined voxels showing BBBD in % of the volume in each of the four compartments was significantly higher for the ABT compartment than for the NBT compartments at a distance of 0–1, 1–2 or 2–3 cm from the ABT compartment. The circle illustrates the four tissue compartments. (b) A similar spatial pattern is observed in all three MRIs. Thus, the absolute BBBD volume in both the ABT compartment and the neighbouring NBT compartment between 0 and 1 cm from the ABT compartment was significantly greater in patients developing post-stroke epilepsy (PSE) than in non-PSE patients. The data suggest that the large early volume of BBBD in the ABT compartment and adjacent NBT compartment in patients who developed PSE was not simply followed by glial scarring in the late phase, but by a structural disturbance characterised by chronic BBBD. ABT, abnormal brain tissue; BBBD, blood–brain barrier dysfunction; CE-MRI, contrast-enhanced magnetic resonance imaging; NBT, normal brain tissue.

To this end, two objects in the vicinity of the ABT were generated based on a distance transform map (DTM), where each NBT voxel was assigned with its Euclidian distance to the closest ABT edge voxel. NBT voxels located at DTM<1cm formed the first in the vicinity of the ABT object and the second and third were 1cm≤DTM<2cm and 2 cm≤DTM<3cm. As reported previously, the three objects were used to quantify amounts of BBB permeability in three ring zones around the ABT (Fig. 3).^41^

### Neuromonitoring analysis

Both SD and electrographic seizures are known to occur after aSAH.^35^^,^^44^ We adhered to the recommendations of the Co-Operative Studies on Brain Injury Depolarisations (COSBID) group for recording, analysing and interpreting SD and electrographic seizures.^38^ For this secondary study, except for a few additions, we utilised the manual analysis of SD and electrographic seizures from the primary analysis of DISCHARGE-1, as previously detailed.^24^ Each recording day for each patient was assessed for the total (cumulative) SD-induced depression duration (TDDD) and the number of SD.^38^ We then calculated peak values for each SD-related variable resulting in peak total SD-induced depression duration (PTDDD) and peak number of SD (peak_SD_) for the recording period. In every patient, the first 24-h period after the initial haemorrhage was always denoted as ‘day 0’, the second 24-h period as ‘day 1’ and so on.^24^

Electrographic seizures were defined as any spikes, sharp-waves, or sharp-and-slow wave complexes lasting for 10s or more at either a frequency of at least 3/s or a frequency of at least 1/s with clear evolution in frequency, morphology, or recording sites of the electrode strip.^38^ These changes manifest as increases in the alternating current (AC)-ECoG power and the integral of the power, which can occur with or without an overt clinical correlate. Total electrographic seizure duration of each recording day (TSDD) and the peak total electrographic seizure duration (PTSDD) were calculated in analogy to the TDDDs and the PTDDD for SD. In addition, the peak number of electrographic seizures (peak_seizure_) was determined similar to peak_SD_.

Overall, ECoG recordings were available in 126 of 130 patients (96.9%) to determine TDDDs, PTDDD, peak_SD_, TSDDs, PTSDD und peak_seizure_ based on 4276 SD in 109/126 patients (86.5%) and 250 electrographic seizures in 11/126 patients (8.7%).

### Diagnosis of post-haemorrhagic epilepsy

For diagnosing PSE, a semi-structured telephone interview was conducted by a neurologist in training, as previously reported.^35^ If the patient was unable to provide a history, relatives or caregivers were interviewed instead. Medical records of subsequent hospitalisations or doctor's visits were also considered when available. The interview began with a screening questionnaire. If the screening criteria were met, a detailed history of seizures was taken, including information on the timing, seizure type, concurrent medical problems and the use of anti-seizure medication. The definition of a seizure adhered to the current guidelines of the International League Against Epilepsy (ILAE).^45^^,^^46^ Seizures within 12 h of the initial haemorrhage were defined as onset seizures. Seizures that occurred between 12 h and 21 days, i.e. during the subsequent in-hospital phase, were defined as subacute seizures. Post-stroke seizures were defined as those occurring after day 21—typically after the patients had been discharged from acute neurocritical care. Following guidelines of the ILAE, a single post-stroke seizure was sufficient for diagnosing structural epilepsy if neuroimaging revealed focal brain damage, which was confirmed in all patients with at least one post-stroke seizure.^45^

### Populations, missing values and estimands

Definitions of populations and outcomes and methods for missing data follow Fig. 1. Of the 242 aSAH patients in the database, six did not meet the inclusion/exclusion criteria, leaving 236 patients. Two patients were excluded *a priori* because they already suffered from epilepsy before aSAH. BBB assessment requires a CE-MRI. Therefore, 104 patients were excluded *a priori* because they had neither undergone CE-MRI_early_ nor CE-MRI_post-monitoring_. Accordingly, the first target population of our analysis consisted of the remaining 130 patients (n = 99 patients with both CE-MRI_early_ and CE-MRI_post-monitoring_, 11 patients with only CE-MRI_early_ and 20 patients with only CE-MRI_post-monitoring_). CE-MRI_early_ was performed on median day 2 (i.e., between 48 and 72 h after the initial haemorrhage, IQR: 1–3) and CE-MRI_post-monitoring_ on median day 15 (IQR: 13–16). CE-MRI_early_ was always performed after aneurysm occlusion (usually between 24 and 48 h after occlusion). Eligible patients who underwent CE-MRI during neurocritical care were enrolled between January 2007 and March 2018 (11.3 years). The primary statistical analysis took place from April to August 2024.

For all 130 patients, multiple imputation was used for missing predictor variables. Nine of 130 patients died in hospital within 21 days of the initial aSAH. Therefore, by definition, they had no chance of developing PSE, and we used the binary endpoint of ’early death’ (yes/no) in this population and excluded these 9 patients from further analysis.

In the analysis of the remaining 121 patients, which is the primary analysis, the target population consisted of all patients who belong to the first target population and additionally survived at least 21 days. Information on PSE was missing for 28 of 121 patients (23.1%). Patients/carers were unreachable in 12 of 28 cases. A further 11 of the 28 patients were confirmed deceased but their PSE status was unknown. In the 121 patients, the combined binary outcome ‘PSE or late death’ was used, including multiple imputation for this outcome for 17 patients (5 + 12). Additionally, missing values of predictors were imputed. For the remaining 93 patients with complete outcome data, we only imputed missing predictor data. We selected this population for a sensitivity analysis only, as no meaningful target population was definable at the time of the prognostic measurements. The same holds for an additional complete case analysis (within the 121 patients of the primary target population) without any imputation of missing values and different population sizes for different predictors.

### Statistics

**Fig. 4:**
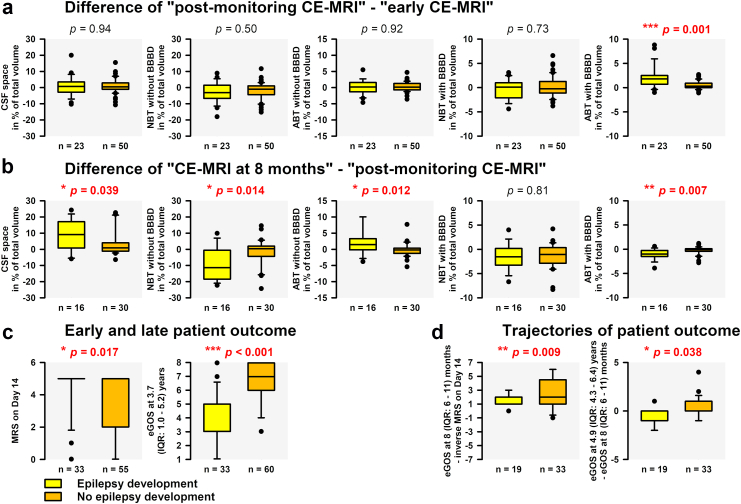
**MRI changes over time**. In all panels, the individual variables are compared between patients who developed post-stroke epilepsy (PSE) and non-PSE patients (Mann–Whitney Rank Sum Tests). (a) The variables examined are the differences obtained by subtracting the respective value in the CE-MRI_early_ from the value in the CE-MRI_post-monitoring_. (b) The variables examined are the differences obtained by subtracting the respective value in the CE-MRI_post-monitoring_ from the value in the CE-MRI_late_. (c) Early (MRS_Day14_) and late (eGOS at 3.7 years) patient outcome. (d) The variables examined are the differences obtained by subtracting the respective outcome value at the first time point from the outcome value at the second time point. Left graph: To subtract MRS_Day14_ (0–6) from eGOS_Month8_ (1–8), MRS_Day14_ (6–0) was first converted to the inverse MRS_Day14_ (1–7). Although the MRS and eGOS scales differ slightly in content, they are sufficiently similar after inverting the MRS scale to estimate the strength of recovery between two points in time based on the trajectory obtained by subtraction. Right graph: While eGOS in patients with PSE tends to worsen over time, non-PSE patients tend to show further improvement. ABT, abnormal brain tissue; BBBD, blood–brain barrier dysfunction; CE-MRI, contrast-enhanced magnetic resonance imaging; CSF, cerebrospinal fluid; eGOS, extended Glasgow Outcome Scale; MRS, Modified Rankin Scale; NBT, normal brain tissue.

For each analysis, binary logistic regression was applied. Model-based event probabilities were entered as predictors in area-under-the-curve (AUC) analyses. The leaving-one-out method was used to estimate the area under the receiver operating characteristic (ROC) curve. In each leaving-one-out sample, variable selection was performed separately and the predicted probability was stored for the one sample left out. This was combined with multiple imputation for the 15 predictor variables (500 imputation samples).^47^ As WFNS and Rosen Macdonald Score (RMS)^39^ were complete, we assumed missing at random of the other data conditional on these initial clinical scores. The level of significance was 0.05 (two-sided). Effect sizes (OR) including two-sided 95% CI are given. Multiplicity was addressed to 15 covariates in the univariate analysis using the Bonferroni approach, thus a raw *p*-value of 0.0033 or lower led to a significant result. No Bonferroni correction was applied with respect to the different outcomes, as only the combined endpoint death (later than 21 days) and/or epilepsy was primary. In multivariate analysis, forward variable selection (*p*_inclusion_ = 0.05, *p*_exclusion_ = 0.10) was applied. Additional analyses for complete case without imputation included *p*-values for non-parametric tests (Mann–Whitney Rank Sum Test (MWRST), Friedman test including pairwise comparisons with Dunn's test) in descriptive analyses (Figs. 3 and 4). Unless otherwise stated, these data are reported as median (IQR). Analyses were conducted using SPSS for Windows (release 28).

In an *ex post* power analysis with 93 patients with information on the PSE status (PSE: 33 patients, no PSE: 60 patients), an effect size of 0.641 could be detected (as different from zero) with a power of 80% in t-tests for independent samples. In the analysis of early death with 9 events and 121 patients without event, the detectable effect size was 0.980.

### Role of funders

Funders did not have any role in study design, data collection, data analyses, interpretation, or writing of report.

## Results

In our initial analysis of 130 patients, we examined the predictive power of the 15 variables collected during neurocritical care for early death within 21 days. We found uncorrected *p*-values <0.05 (simple logistic regression analysis, Wald χ^2^ test for each variable) for ABT_iBBB_%_early_ (OR: 1.246, 95% CI: 1.013–1.532, *p* = 0.037), ABT_BBBD_%_early_ (OR: 2.523, 95% CI: 1.099–5.789, *p* = 0.029), PTDDD (OR: 7.464, 95% CI: 1.570–35.482, *p* = 0.011), peak_SD_ (OR: 20.973, 95% CI: 2.058–213.744, *p* = 0.010), NBT_iBBB_%_post-monitoring_ (OR: 0.898, 95% CI: 0.826–0.976, *p* = 0.012), ABT_iBBB_%_post-monitoring_ (OR: 1.721, 95% CI: 1.224–2.419, *p* = 0.002) and ABT_BBBD_%_post-monitoring_ (OR 1.619, 95% CI: 1.228–2.133, *p* = 0.001) (Table 2). Thus, only the variables ABT_iBBB_%_post-monitoring_ (*p* = 0.002) and ABT_BBBD_%_post-monitoring_ (*p* = 0.001) fulfilled the criterion of significance after Bonferroni correction (*p* < 0.0033). However, there was a lack of power due to the small number of 9 events. In multivariate analysis (multiple logistic regression analysis, Wald χ^2^ test) with forward variable selection, only ABT_BBBD_%_post-monitoring_ (OR: 1.619, 95% CI: 1.228–2.133, *p* = 0.001) remained significant. The AUC was 0.866 (95% CI: 0.738–0.993) with leaving-one-out correction.

**Table 2:** Analysis of n = 121 early survivors versus n = 9 patients who died within 21 days.

Variable	Regression coefficient	Standard error	*p-*value	Odds ratio	95% confidence interval
WFNS	0.518	0.289	0.073	1.678	0.953–2.954
RMS	0.301	0.186	0.107	1.351	0.938–1.946
NBT_iBBB_%_early_	−0.048	0.042	0.25	0.953	0.878–1.034
*ABT*_*iBBB*_*%*_*early*_	*0.220*	*0.105*	*0.037*	*1.246*	*1.013–1.532*
NBT_BBBD_%_early_	0.229	0.165	0.166	1.257	0.910–1.738
*ABT*_*BBBD*_*%*_*early*_	*0.925*	*0.424*	*0.029*	*2.523*	*1.099–5.789*
PTSDD [min]	0.682	0.600	0.26	1.978	0.610–6.417
Peak_seizure_	1.257	0.745	0.091	3.516	0.817–15.136
*PTDDD [min]*	*2.010*	*0.795*	*0.011*	*7.464*	*1.570–35.482*
*Peak*_*SD*_	*3.043*	*1.184*	*0.010*	*20.973*	*2.058–213.744*
MRS_Day14_	23.465	2148.991	0.99	n.a.	n.a.
*NBT*_*iBBB*_*%*_*post-monitoring*_	*−0.107*	*0.043*	*0.012*	*0.898*	*0.826–0.976*
*ABT*_*iBBB*_*%*_*post-monitoring*_	*0.543*	*0.174*	*0.002*	*1.721*	*1.224–2.419*
NBT_BBBD_%_post-monitoring_	0.067	0.133	0.62	1.069	0.823–1.388
*ABT*_*BBBD*_*%*_*post-monitoring*_	*0.482*	*0.141*	*0.001*	*1.619*	*1.228–2.133*

Variables with uncorrected *p*-values <0.05 are marked in italic. ABT_BBBD_%_early_, abnormal brain tissue with blood–brain barrier dysfunction as a percentage of total intracranial volume in the early MRI; ABT_BBBD_%_post-monitoring_, abnormal brain tissue with blood–brain barrier dysfunction as a percentage of total intracranial volume in the post-monitoring MRI; ABT_iBBB_%_early_, abnormal brain tissue with intact blood–brain barrier as a percentage of total intracranial volume in the early MRI; ABT_iBBB_%_post-monitoring_, abnormal brain tissue with intact blood–brain barrier as a percentage of total intracranial volume in the post-monitoring MRI; MRS_Day14_, Modified Rankin Scale post monitoring; n. a., not applicable (did not converge); NBT_BBBD_%_early_, normal brain tissue with blood–brain barrier dysfunction as a percentage of total intracranial volume in the early MRI; NBT_BBBD_%_post-monitoring_, normal brain tissue with blood–brain barrier dysfunction as a percentage of total intracranial volume in the post-monitoring MRI; NBT_iBBB_%_early_, normal brain tissue with intact blood–brain barrier as a percentage of total intracranial volume in the early MRI; NBT_iBBB_%_post-monitoring_, normal brain tissue with intact blood–brain barrier as a percentage of total intracranial volume in the post-monitoring MRI; peak_SD_, peak number of spreading depolarisations of a recording day; peak_seizure_, peak number of electrographic seizures of a recording day; PTSDD, peak total electrographic seizure duration of a recording day; PTDDD, peak total spreading depolarisation-induced depression duration of a recording day; RMS, Rosen Macdonald Score^39^; WFNS, World Federation of Neurosurgical Societies score. Nine of 130 patients (6.9%) died within 21 days on median day 18 (interquartile range (IQR): 11–19). In all cases, the MRS value was determined either on day 14 or on the day immediately before death if death occurred before day 15. Seven of the 9 patients who died early had a contrast-enhanced (CE)-MRI_post-monitoring_ on median day 11 (IQR: 7–15; range: 6–18).

The 121 study subjects of the primary analysis are demographically described in Table 3. In the primary analysis with outcome ‘PSE or late death’, the following variables revealed uncorrected *p*-values <0.05 (simple logistic regression analysis, Wald χ^2^ test for each variable): WFNS (OR: 1.383, 95% CI: 1.041–1.838, *p* = 0.025), RMS (OR: 1.294, 95% CI: 1.036–1.617, *p* = 0.023), NBT_BBBD_%_early_ (OR: 1.255, 95% CI: 1.024–1.537, *p* = 0.028), ABT_BBBD_%_early_ (OR: 2.128, 95% CI: 1.083–4.182, *p* = 0.028), MRS_Day14_ (OR: 1.490, 95% CI: 1.088–2.038, *p* = 0.013), NBT_iBBB_%_post-monitoring_ (OR: 0.940, 95% CI: 0.887–0.996, *p* = 0.038), ABT_iBBB_%_post-monitoring_ (OR: 1.170, 95% CI: 1.007–1.358, *p* = 0.040) and ABT_BBBD_%_post-monitoring_ (OR: 2.090, 95% CI: 1.373–3.183, *p* = 0.001) (Table 4). Thus, only ABT_BBBD_%_post-monitoring_ remained significant after Bonferroni correction. This was also the only variable left in a multivariate analysis with forward selection (multiple logistic regression analysis, Wald χ^2^ test). The AUC was 0.733 (95% CI: 0.590–0.875) after leaving-one-out correction.

**Table 3:** Summary of demographic, treatment, monitoring and outcome data of the 121 patients in the primary analysis.

Variable	Median	First quartile	Third quartile	n
Age	55	48	63	121
WFNS	3	1	5	121
RMS	6	4	7	121
ECoG recording time [days]	9.7	8.3	11.5	117
PTSDD [min]	0.0	0.0	0.0	117
Peak_seizure_	0.0	0.0	0.0	117
PTDDD [min]	106.9	27.4	350.9	117
Peak_SD_	7.0	3.0	18.9	117
DSA grading score	1.3	1.1	1.7	100
TCD-determined peak_mbfv_ [cm/s]	146	140	213	115
MRS_Day14_	5	3	5	116
eGOS at 3.2 (IQR: 0.6–4.9) years	6	3	7	109

Variable	1st category	2nd category	Ratio	Unknown
Sex	Females: n = 79 (65.3%)	Males: n = 42 (34.7%)	1.9: 1.0^a^	–
Family history of epilepsy	Yes: n = 4 (3.3%)	No: n = 94 (77.7%)	1.0: 23.5^b^	n = 23 (19.0%)
MFS	1 or 2: n = 9 (7.4%)	3 or 4: n = 112 (92.6%)	1.0–12.4	–
Onset seizure	Yes: n = 36 (29.8%)	No: n = 71 (58.7%)	1.0: 2.0	n = 14 (11.6%)
Procedure for occluding the aneurysm	Clipping: n = 108 (89.3%)	Coilng: n = 13 (10.7%)	8.3: 1.0	–
Oral nimodipine prophylaxis	Yes: n = 109 (90.1)	No: n = 12 (9.9)	9.1: 1.0	
Electrographic seizures during neuromonitoring	Yes: n = 10 (8.3%)	No: n = 107 (88.4%)	1.0: 10.7	n = 4 (3.3%)
SD during neuromonitoring	Yes: n = 101 (83.5%)	No: n = 16 (13.2%)	6.3: 1.0	n = 4 (3.3%)
PSE	Yes: n = 33 (27.3%)	No: n = 60 (49.6%)	1.8: 1.0	n = 28 (23.1%)
Late death	Yes: n = 16 (13.2%)	No: n = 93 (76.9%)	5.8: 1.0	n = 12 (9.9%)

DSA, digital subtraction angiography; ECoG, electrocorticography; eGOS, extended Glasgow Outcome Scale; MFS, Modified Fisher Scale^48^; MRS_Day14_, Modified Rankin Scale post monitoring; peak_mbfv_, median of the two peak velocities determined separately for each of the two middle cerebral arteries by transcranial Doppler-sonography (TCD) during the monitoring period; peak_SD_, peak number of spreading depolarisations of a recording day; peak_seizure_, peak number of electrographic seizures of a recording day; PSE, post-stroke epilepsy; PTSDD, peak total electrographic seizure duration of a recording day; PTDDD, peak total spreading depolarisation-induced depression duration of a recording day; RMS, Rosen Macdonald Score^39^; SD, spreading depolarisation; WFNS, World Federation of Neurosurgical Societies score.

a The female to male ratio of 1.9:1.0 aligns with the typical female to male ratio of 1.8:1.0 for aneurysmal subarachnoid haemorrhage (aSAH) in Germany.^27^

b None of the patients with a family history of epilepsy developed PSE.

**Table 4:** Primary analysis of n = 121 patients (outcome: death later than 21 days after the initial haemorrhage and/or epilepsy).

Variable	Regression coefficient	Standard error	*p-*value	Odds ratio	95% confidence interval
WFNS	0.324	0.145	0.025	1.383	1.041–1.838
*RMS*	*0.258*	*0.114*	*0.023*	*1.294*	*1.036–1.617*
NBT_iBBB_%_early_	−0.041	0.035	0.24	0.960	0.897–1.028
ABT_iBBB_%_early_	0.116	0.095	0.22	1.123	0.932–1.354
*NBT*_*BBBD*_*%*_*early*_	*0.227*	*0.104*	*0.028*	*1.255*	*1.024–1.537*
*ABT*_*BBBD*_*%*_*early*_	*0.755*	*0.345*	*0.028*	*2.128*	*1.083–4.182*
PTSDD [min]	−0.082	0.556	0.88	0.921	0.310–2.738
Peak_seizure_	0.018	0.941	0.98	1.019	0.161–6.447
PTDDD [min]	0.224	0.241	0.35	1.252	0.780–2.009
Peak_SD_	0.136	0.426	0.75	1.145	0.497–2.637
*MRS*_*Day14*_	*0.398*	*0.160*	*0.013*	*1.490*	*1.088–2.038*
*NBT*_*iBBB*_*%*_*post-monitoring*_	*−0.062*	*0.030*	*0.038*	*0.940*	*0.887–0.996*
*ABT*_*iBBB*_*%*_*post-monitoring*_	*0.157*	*0.076*	*0.040*	*1.170*	*1.007–1.358*
NBT_BBBD_%_post-monitoring_	0.165	0.087	0.059	1.179	0.994–1.398
*ABT*_*BBBD*_*%*_*post-monitoring*_	*0.737*	*0.215*	*0.001*	*2.090*	*1.373–3.183*

Variables with uncorrected *p*-values <0.05 are marked in italic. ABT_BBBD_%_early_, abnormal brain tissue with blood–brain barrier dysfunction as a percentage of total intracranial volume in the early MRI; ABT_BBBD_%_post-monitoring_, abnormal brain tissue with blood–brain barrier dysfunction as a percentage of total intracranial volume in the post-monitoring MRI; ABT_iBBB_%_early_, abnormal brain tissue with intact blood–brain barrier as a percentage of total intracranial volume in the early MRI; ABT_iBBB_%_post-monitoring_, abnormal brain tissue with intact blood–brain barrier as a percentage of total intracranial volume in the post-monitoring MRI; MRS_Day14_, Modified Rankin Scale post monitoring; n. a., not applicable (did not converge); NBT_BBBD_%_early_, normal brain tissue with blood–brain barrier dysfunction as a percentage of total intracranial volume in the early MRI; NBT_BBBD_%_post-monitoring_, normal brain tissue with blood–brain barrier dysfunction as a percentage of total intracranial volume in the post-monitoring MRI; NBT_iBBB_%_early_, normal brain tissue with intact blood–brain barrier as a percentage of total intracranial volume in the early MRI; NBT_iBBB_%_post-monitoring_, normal brain tissue with intact blood–brain barrier as a percentage of total intracranial volume in the post-monitoring MRI; peak_SD_, peak number of spreading depolarisations of a recording day; peak_seizure_, peak number of electrographic seizures of a recording day; PTSDD, peak total electrographic seizure duration of a recording day; PTDDD, peak total spreading depolarisation-induced depression duration of a recording day; RMS, Rosen Macdonald Score^39^; WFNS, World Federation of Neurosurgical Societies score.

Among the 104 patients with complete data on the combined binary endpoint ‘PSE or late death’, 26 of the 68 women (38.2%) showed this endpoint, compared with 18 of the 36 men (50.0%, *p* = 0.25, Pearson χ^2^ test). Among the 93 patients with complete data on PSE and late death, 33 (35.5%) developed PSE. Five of 33 patients with PSE (15.2%) and 0/60 patients without PSE died. In the first sensitivity analysis of the 93 patients with available outcome but imputation of predictors, the AUC was 0.722 (95% CI: 0.593–0.850) after leaving-one-out correction (Table 5). In complete case analysis without imputation, the AUC was 0.713 (95% CI: 0.659–0.829) after leaving-one-out correction (Table 6). In both analyses, ABT_BBBD_%_post-monitoring_ was the only significant variable (multiple logistic regression analysis, Wald χ^2^ test).

**Table 5:** Sensitivity analysis (n = 33 patients who developed late epilepsy compared to n = 60 patients who did not develop epilepsy).

Variable	Regression coefficient	Standard error	*p-*value	Odds ratio	95% confidence interval
WFNS	0.319	0.150	0.033	1.376	1.025–1.847
*RMS*	*0.232*	*0.114*	*0.042*	*1.261*	*1.008–1.576*
NBT_iBBB_%_early_	−0.044	0.036	0.22	0.957	0.892–1.026
ABT_iBBB_%_early_	0.130	0.101	0.196	1.139	0.935–1.387
*NBT*_*BBBD*_*%*_*early*_	*0.243*	*0.109*	*0.025*	*1.275*	*1.030–1.579*
*ABT*_*BBBD*_*%*_*early*_	*0.822*	*0.371*	*0.027*	*2.275*	*1.100–4.706*
PTSDD [min]	−0.008	0.559	0.99	0.992	0.332–2.964
Peak_seizure_	0.107	0.946	0.91	1.113	0.174–7.105
PTDDD [min]	0.230	0.260	0.38	1.258	0.756–2.094
Peak_SD_	0.098	0.454	0.83	1.103	0.453–2.684
*MRS*_*Day14*_	*0.355*	*0.158*	*0.025*	*1.426*	*1.046–1.944*
NBT_iBBB_%_post-monitoring_	−0.058	0.031	0.058	0.943	0.888–1.002
ABT_iBBB_%_post-monitoring_	0.121	0.081	0.137	1.128	0.963–1.322
NBT_BBBD_%_post-monitoring_	0.162	0.091	0.074	1.176	0.984–1.406
*ABT*_*BBBD*_*%*_*post-monitoring*_	*0.758*	*0.218*	*0.001*	*2.134*	*1.392–3.271*

Variables with uncorrected *p*-values <0.05 are marked in italic. ABT_BBBD_%_early_, abnormal brain tissue with blood–brain barrier dysfunction as a percentage of total intracranial volume in the early MRI; ABT_BBBD_%_post-monitoring_, abnormal brain tissue with blood–brain barrier dysfunction as a percentage of total intracranial volume in the post-monitoring MRI; ABT_iBBB_%_early_, abnormal brain tissue with intact blood–brain barrier as a percentage of total intracranial volume in the early MRI; ABT_iBBB_%_post-monitoring_, abnormal brain tissue with intact blood–brain barrier as a percentage of total intracranial volume in the post-monitoring MRI; MRS_Day14_, Modified Rankin Scale post monitoring; n. a., not applicable (did not converge); NBT_BBBD_%_early_, normal brain tissue with blood–brain barrier dysfunction as a percentage of total intracranial volume in the early MRI; NBT_BBBD_%_post-monitoring_, normal brain tissue with blood–brain barrier dysfunction as a percentage of total intracranial volume in the post-monitoring MRI; NBT_iBBB_%_early_, normal brain tissue with intact blood–brain barrier as a percentage of total intracranial volume in the early MRI; NBT_iBBB_%_post-monitoring_, normal brain tissue with intact blood–brain barrier as a percentage of total intracranial volume in the post-monitoring MRI; peak_SD_, peak number of spreading depolarisations of a recording day; peak_seizure_, peak number of electrographic seizures of a recording day; PTSDD, peak total electrographic seizure duration of a recording day; PTDDD, peak total spreading depolarisation-induced depression duration of a recording day; RMS, Rosen Macdonald Score^39^; WFNS, World Federation of Neurosurgical Societies score.

**Table 6:** Complete case analysis of n = 130 patients (outcome: death later than 21 days after the initial haemorrhage and/or epilepsy, leaving one out correction).

Variable	Regression coefficient	Standard error	*p-*value	Odds ratio	95% confidence interval	Missing cases
*WFNS*	*0.338*	*0.149*	*0.023*	*1.402*	*1.047–1.879*	*27*
*RMS*	*0.250*	*0.113*	*0.027*	*1.283*	*1.029–1.601*	*27*
NBT_iBBB_%_early_	−0.048	0.039	0.21	0.953	0.883–1.028	41
ABT_iBBB_%_early_	0.192	0.107	0.072	1.212	0.983–1.494	41
*NBT*_*BBBD*_*%*_*early*_	*0.257*	*0.112*	*0.022*	*1.293*	*1.038–1.611*	*41*
*ABT*_*BBBD*_*%*_*early*_	*1.009*	*0.373*	*0.007*	*2.743*	*1.321–5.698*	*41*
PTSDD [min]	−0.003	0.556	1.00	0.997	0.335–2.965	29
Peak_seizure_	0.132	0.943	0.89	1.141	0.180–7.241	29
PTDDD [min]	0.296	0.259	0.25	1.344	0.809–2.232	29
Peak_SD_	0.221	0.450	0.62	1.247	0.516–3.013	29
*MRS*_*Day14*_	*0.369*	*0.159*	*0.020*	*1.447*	*1.060–1.975*	*32*
NBT_iBBB_%_post-monitoring_	−0.054	0.031	0.085	0.947	0.891–1.077	33
ABT_iBBB_%_post-monitoring_	0.121	0.084	0.147	1.129	0.958–1.331	33
NBT_BBBD_%_post-monitoring_	0.174	0.092	0.058	1.191	0.994–1.426	33
*ABT*_*BBBD*_*%*_*post-monitoring*_	*0.800*	*0.220*	*<0.001*	*2.225*	*1.446–3.422*	*33*

Variables with uncorrected *p* values < 0.05 are marked in italic. ABT_BBBD_%_early_, abnormal brain tissue with blood–brain barrier dysfunction as a percentage of total intracranial volume in the early MRI; ABT_BBBD_%_post-monitoring_, abnormal brain tissue with blood–brain barrier dysfunction as a percentage of total intracranial volume in the post-monitoring MRI; ABT_iBBB_%_early_, abnormal brain tissue with intact blood–brain barrier as a percentage of total intracranial volume in the early MRI; ABT_iBBB_%_post-monitoring_, abnormal brain tissue with intact blood–brain barrier as a percentage of total intracranial volume in the post-monitoring MRI; MRS_Day14_, Modified Rankin Scale post monitoring; NBT_BBBD_%_early_, normal brain tissue with blood–brain barrier dysfunction as a percentage of total intracranial volume in the early MRI; NBT_BBBD_%_post-monitoring_, normal brain tissue with blood–brain barrier dysfunction as a percentage of total intracranial volume in the post-monitoring MRI; NBT_iBBB_%_early_, normal brain tissue with intact blood–brain barrier as a percentage of total intracranial volume in the early MRI; NBT_iBBB_%_post-monitoring_, normal brain tissue with intact blood–brain barrier as a percentage of total intracranial volume in the post-monitoring MRI; peak_SD_, peak number of spreading depolarisations of a recording day; peak_seizure_, peak number of electrographic seizures of a recording day; PTSDD, peak total electrographic seizure duration of a recording day; PTDDD, peak total spreading depolarisation-induced depression duration of a recording day; RMS, Rosen Macdonald Score^39^; WFNS, World Federation of Neurosurgical Societies score.

### ABT_BBBD_%_post-monitoring_, ABT_iBBB_%_post-monitoring_ and PTDDD

To better understand the results, we further analysed ABT%_post-monitoring_ and PTDDD, the strongest neuroimaging and the strongest ECoG predictor of mortality in the post-monitoring phase in DISCHARGE-1.^24^ As in DISCHARGE-1, these two variables were also strong predictors of mortality in the complete case analysis without imputation in the present study (outcome: early or late death after the neuromonitoring phase, AUC of ABT%_post-monitoring_: 0.852 (95% CI: 0.761–0.942, *p* < 0.0001, n = 108) and AUC of PTDDD: 0.784 (95% CI: 0.674–0.895), *p* < 0.0001, n = 115). In addition, CE-MRIs enabled the two components of ABT%_post-monitoring_, namely ABT_BBBD_%_post-monitoring_ and ABT_iBBB_%_post-monitoring_, to be considered separately in the present study. Supplementary Figure S1 shows that both components were strongly correlated with each other and with PTDDD, but PTDDD was more strongly correlated with ABT_iBBB_%_post-monitoring_ than with ABT_BBBD_%_post-monitoring_. As expected, these correlations were slightly stronger for the ipsilateral hemisphere (Supplementary Figure S1a, c, e) where the electrode strip was positioned than for the entire brain (Supplementary Figure S1b, d, f). Supplementary Figure S2 visually illustrates that all three variables, namely ABT_BBBD_%_post-monitoring_, ABT_iBBB_%_post-monitoring_, and PTDDD, were strong predictors of mortality in the post-monitoring phase, but only ABT_BBBD_%_post-monitoring_ was a significant predictor of epileptogenesis.

### Spatial analysis of BBBD

Fig. 3 describes BBBD in temporal and spatial context based on the 93 patients with available outcome for PSE (complete case analysis without imputation). In all three CE-MRIs, CE-MRI_early_, CE-MRI_post-monitoring_ and CE-MRI_late_ at median month 8 (IQR: 6–10), the BBBD volume as a percentage of the respective tissue volume was higher in ABT than in NBT at a distance of 0–1, 1–2, or 2–3 cm from the ABT (Fig. 3a). Not only in the ABT zone, but also in the NBT zone directly adjacent to ABT, i.e. at a distance between 0 and 1 cm from the ABT zone, the absolute BBBD volume was greater in all three CE-MRI scans in patients with PSE than in patients without PSE (Fig. 3b).

### Temporal changes in CE-MRI variables

**Fig. 5:**
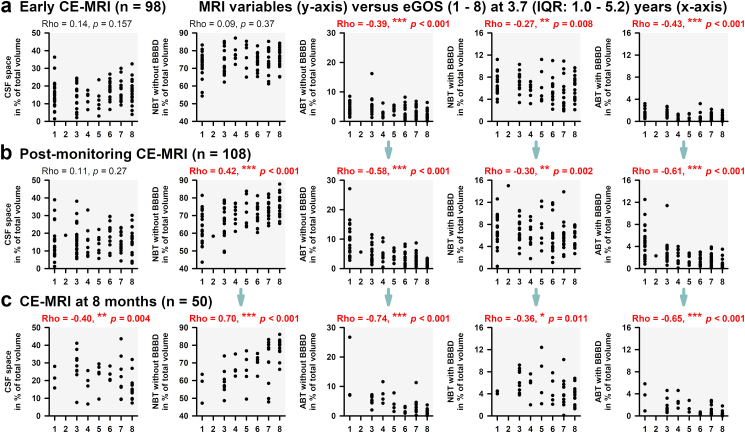
**CE-MRI and patient outcome**. For each of the three CE-MRI scans (a–c), all five MRI variables are plotted against the patient outcome 3.7 years after the initial haemorrhage. The Spearman correlation coefficients (SC) increased for all five MRI variables examined with each CE-MRI. The pattern that emerges is that the development of brain atrophy, gliosis, and enlarged CSF spaces was more pronounced the poorer the patient outcome. ABT, abnormal brain tissue; BBBD, blood–brain barrier dysfunction; CE-MRI, contrast-enhanced magnetic resonance imaging; CSF, cerebrospinal fluid; eGOS, extended Glasgow Outcome Scale; MRS, Modified Rankin Scale; NBT, normal brain tissue; Rho, Spearman's Rho.

Fig. 4 describes the changes over time from CE-MRI_early_ to CE-MRI_post-monitoring_ and from CE-MRI_post-monitoring_ to CE-MRI_late_ in patients with two consecutive MRIs and available outcome for PSE (complete case analysis without imputation). For the period between CE-MRI_early_ and CE-MRI_post-monitoring_, ABT_BBBD_% increased in patients with PSE compared to patients without PSE, and then decreased between CE-MRI_post-monitoring_ and CE-MRI_late_. Despite this late decline, patients with PSE continued to show higher ABT_BBBD_% than patients without PSE in CE-MRI_late_ (1.3 (IQR: 0.9–2.6) versus 0.5 (0.2–1.2) %, MWRST, *p* = 0.003). These changes went along with brain atrophy in patients with PSE, reflected by greater decrease of NBT_iBBB_% and increase of internal and external CSF spaces from CE-MRI_post-monitoring_ to CE-MRI_late_. ABT_iBBB_% also increased which most closely reflects gliosis at this late stage. Overall, NBT_iBBB_%_late_ was substantially lower in patients with PSE than in patients without PSE (63.5 (IQR: 56.4–70.0) versus 75.3 (66.0–80.3) %, MWRST, *p* = 0.001). Fig. 4c shows that patients with PSE had worse clinical outcome. A complete set of MRS_Day14_, early extended Glasgow Outcome Scale (eGOS) at 8 (IQR: 6–11) months and late eGOS was available in 52 patients (Fig. 4d). The trajectory of these scores suggests that the initial recovery of patients with PSE was weaker, similar to previous observations,^30^ and that they were more likely to deteriorate again in the long term, whereas patients without PSE tended to improve. When examining the progression from CE-MRI_early_ to CE-MRI_post-monitoring_ to CE-MRI_late_, the correlation strengths with the last eGOS at 3.7 (1.0–5.2) years increased for all MRI variables; i.e., from the first to the third MRI, the increasing development of brain atrophy, gliosis, and enlarged CSF spaces was associated with poor patient outcomes (Fig. 5).

## Discussion

Our analyses addressed long-term outcome prediction after aSAH using variables available during neurocritical care. Based on previous experimental evidence,^49^ we prioritised CE-MRI–derived BBB metrics in this translational study; while subdural ECoG was recorded per COSBID protocols,^38^ scalp EEG was not systematically acquired across centres. Histopathology was not available in this clinical cohort.

First, we assessed predictive variables for early death defined by day 21. ABT_BBBD_%_post-monitoring_ was the only independent predictor, but the power was very small due to the small number of 9 deaths. Thus, we note that the two neuromonitoring variables PTDDD and peak_SD_ were also univariately significant (without Bonferroni correction). Second, we examined predictors for the combined endpoint ‘PSE or late death’ in patients who survived the first 21 days. In these patients, the same MRI variable, ABT_BBBD_%_post-monitoring_, which independently predicted early death was also the only independent predictor of ‘PSE or late death’. This result, which was obtained by a combination of imputation and the leaving-one-out method, was confirmed in two sensitivity analyses within smaller populations and with fewer missing values.

In the DISCHARGE-1 trial, ABT%_post-monitoring_ in the hemisphere ipsilateral to the subdural electrodes was previously associated in multiple regression with PTDDD (*β* = 0.413, *p* < 0.001) and the median Glasgow Coma Score value (*β* = −0.306, *p* < 0.001).^24^ This model explained 34% of variance in ABT%_post-monitoring_. In simple regression, PTDDD alone explained 26% of variance in ABT%_post-monitoring_. ABT_BBBD_%_post-monitoring_ and ABT_iBBB_%_post-monitoring_ are the two components that make up ABT%_post-monitoring_. In the present study, we found that although PTDDD correlated with both components, it was more strongly correlated with ABT_iBBB_%_post-monitoring_ than with ABT_BBBD_%_post-monitoring_ (Supplementary Figure S1). ABT_BBBD_%_post-monitoring_, ABT_iBBB_%_post-monitoring_, and PTDDD, were all strong predictors of mortality in the post-monitoring phase, but only ABT_BBBD_%_post-monitoring_ was a significant predictor of epileptogenesis (Supplementary Figure S2).

Mechanistically, subarachnoid blood products and iron deposits as well as SD were reported to trigger endothelial and perivascular inflammation, upregulation of matrix metalloproteinase-9 (MMP-9) and BBBD.^50^, ^51^, ^52^, ^53^, ^54^, ^55^, ^56^, ^57^, ^58^, ^59^ Subsequent serum protein extravasation—most notably albumin—activates astrocytic TGFβ signalling^11^ and disrupts ion/glutamate homoeostasis lowering seizure threshold.^60^ The transformed astrocytes further promote neuroinflammation,^61^ changes in the extracellular matrix^62^ and pathological plasticity,^63^ thereby further contributing to epileptogenesis. BBBD and neuroinflammation reinforce each other, perpetuating network hyperexcitability and progressive tissue injury.^9^^,^^11^^,^^13^, ^14^, ^15^^,^^49^^,^^64^^,^^65^ Experimental and clinical data show that both BBBD and neuroinflammation begin within hours of the initial haemorrhage and continue in the delayed phase.^56^^,^^58^^,^^66^, ^67^, ^68^, ^69^, ^70^, ^71^, ^72^, ^73^, ^74^ The present study shows that BBBD is not completely resolved even 8 months after aSAH (Fig. 3). Whether the resolution of neuroinflammation after aSAH can also be incomplete is the subject of current research.^75^

In DISCHARGE-1, SD variables were included in each multiple regression model for longitudinal neuroimaging-proven early, delayed, and total brain damage, outcome at 7 months, and mortality.^24^ In line with this, PTDDD was also a significant predictor of mortality in the present population (Supplementary Figure S2e), which largely overlaps with the DISCHARGE-1 population. In addition, there is experimental evidence that SD increase BBB permeability.^53^^,^^76^ It may therefore seem paradoxical that, in the present study, SD variables during neurocritical care were not a predictor of epileptogenesis, in contrast to ABT_BBBD_%_post-monitoring_ (Supplementary Figure S2). It is interesting to note in this context that a short-term anti-seizure effect of SD is also being discussed^77^ and that the relationship between SD and BBBD may not be straightforward. For example, a recent study using a photothrombosis model in rats showed that a higher number of SD was associated with a higher lesion volume and more neuronal damage despite shorter electrographic seizures, and that BBB permeability was inversely proportional to the SD load.^78^ SD are discussed to have countless consequences, some of which may be pro-epileptogenic and others anti-epileptogenic,^73^ so that overall, no statistical association between SD and epileptogenesis may emerge, even though SD are clearly associated with tissue damage and mortality.

The observed long-term clinical courses suggest that PSE is a neurodegenerative condition (Fig. 4). This is further reflected in the significant decrease in NBT_iBBB_%, i.e. brain atrophy, with a simultaneous increase in gliosis (ABT_iBBB_%) and enlargement of the CSF spaces in the serial MRIs of patients with PSE compared to patients without PSE. These results are in line with experimental evidence suggesting that BBBD plays a causal role in neurodegeneration.^17^^,^^18^^,^^79^ In this regard, the spatial CE-MRI analysis is interesting, since not only the BBBD volume in the ABT compartment, but also the BBBD volume in the adjacent normal tissue, i.e. between 0 and 1 cm from the ABT, was larger in patients with PSE than in patients without PSE and this remained so even in the CE-MRI after 8 months (Fig. 3).

### Limitations

Our study, a secondary analysis of a prospective, non-interventional, prognostic cohort, has limitations. Because epilepsy was classified as aSAH-related structural PSE without subtyping by seizure semiology, our findings establish the prognostic value of BBBD for aSAH-related ‘PSE or late death’ and may not apply to other stroke aetiologies or epilepsy subtypes.

The requirement of CE-MRI to assess BBBD led to the exclusion of 104 patients who had not undergone CE-MRI during neurocritical care. The reasons for not performing CE-MRI scans varied (Fig. 1) with the most common being borderline haemodynamic stability. The extended duration required for contrast administration necessitated making CE-MRI optional for patient safety, but this resulted in a potential selection bias, as sicker patients were often excluded (Fig. 1).

On the other hand, the patient cohort represented in our database exhibited more severe aSAH than typically reported in other studies.^24^^,^^37^ This is attributable to the fact that the study neurosurgeons opted for invasive neuromonitoring when they assessed a high risk of secondary deterioration. Consequently, the findings from the present study are most applicable to a moderately affected aSAH population. In line with this is the moderate long-term mortality of this cohort (25/130 patients, 19.2%).^29^

Additionally, for 11 of 16 patients who experienced late death, it was not possible to determine the PSE status. However, this did not undermine the primary analysis, which examined the combined endpoint ‘PSE or late death’.

In addition, PSE ascertainment by structured interview and chart review may introduce recall and information bias; however, use of ILAE-concordant criteria and corroborating records mitigates this. Because any misclassification is unlikely to depend on the CE-MRI–derived variables, the net effect would be to attenuate associations toward the null.

The rate of epileptic seizures during neuromonitoring was rather low compared to other studies with invasive neuromonitoring.^80^^,^^81^ This could be due to the fact that patients in our cohort were sedated with propofol and/or midazolam for an average of 9 ± 5 days, i.e. for a relatively long period of time (see Supplementary Figure S4K and L in (24)). Both drugs have a strong anti-seizure effect.

A related aspect is that propofol enhanced BBB permeability in animal studies, whereas midazolam, in contrast, mitigated BBBD.^82^^,^^83^ Speculatively, sedatives could therefore act as modifiers of BBBD. However, the observation that the absolute BBBD volume in the ABT zone and the immediately adjacent NBT zone was greater in all three CE-MRI scans in patients with PSE than in patients without PSE, i.e. even without sedatives in the MRI after 8 months (Fig. 3), argues against the presence of sedatives being a necessary condition for the association between BBBD and epileptogenesis.

Our results point to a worsening clinical condition associated with brain atrophy in patients with PSE compared with patients without PSE. Whether this is actually the manifestation of a progressive neurodegenerative disease would have to be further investigated with serial neuropsychological assessments and longer study duration.

It should also be noted that there are currently no known therapies targeting BBBD or related neuroinflammatory pathways that have demonstrated efficacy in randomised intervention trials for the prevention of epileptogenesis, neurodegeneration or mortality after aSAH. Although our study is the first to show an association of BBBD with ‘PSE or late death” in a clinical cohort, it does not prove a causal relationship, as this would require randomised interventional trials.

Finally, our study included patients from multiple centres, and we acknowledge that variability between centres and, in particular, differences in scanners may have influenced the results. To minimise variability between centres, we implemented several standardisation measures: MRI acquisition protocols, clinical management practices, centralised image processing, and spatial standardisation (all images were spatially normalised to MNI standard space using SPM12 Matlab, correcting for differences in head positioning, orientation, and voxel size across scanners, and ensuring anatomical correspondence across all subjects). As no reference technique exists that measures the same quantities, a direct validation of semi-automatically segmented MRI variables such as ABT_BBBD_%_post-monitoring_, ABT_iBBB_%_post-monitoring_, NBT_BBBD_%_post-monitoring_, and NBT_iBBB_%_post-monitoring_ was not possible. However, one way of indirect validation is to correlate the semi-automatically segmented MRI variables with the manually segmented volume of tissue damage due to ICH, ECI and DCI to determine whether the results are plausible. As shown in Supplementary Figure S3, a particularly strong correlation was found between ABT_BBBD_%_post-monitoring_ and manually segmented tissue damage, which is plausible. Supplementary Figure S4 shows the comparison of these two variables between the different study centres, suggesting that the multicentre effect was rather insignificant.

### Conclusion

ABT_BBBD_%_post-monitoring_ emerged as the sole independent prognostic biomarker for ‘PSE or late death’ among patients with aSAH who survived beyond three weeks. This early biomarker is notably suitable for precision medicine applications as it can be automatically assessed through CE-MRI at the end of the neurocritical care phase. Subsequent CE-MRIs could potentially utilise ABT_BBBD_% to directly measure the effectiveness of pharmacological treatments targeting BBBD and associated pathologies. ABT_BBBD_% may even serve as a pharmacodynamic biomarker, aiding in the optimisation of treatment parameters like dosage and duration, particularly when the therapeutic target involves modulating BBB integrity^8^^,^^10^ or related neuroinflammatory pathways.^73^^,^^75^ Therefore, our findings underscore the value of automated MRI segmentation technology and provide a foundation for further studies to enhance precision medicine strategies, as well as to forge a clear clinical pathway for integrating such innovations into patient care.

## Contributors

JPD and AF had full access to all of the data in the study and take responsibility for the integrity of the data and the accuracy of the data analysis. JPD, PM and AF developed the study concept and design. SL developed the software for the semi-automated segmentation of the MRIs. SL, VH, SM, CLL, VK, MKLW, EJK, KS, NH, AM, ES, JP and CMK were involved in data acquisition, analysis, and interpretation. JPD, PM and AF drafted the manuscript. JPD, SL, OWS, EG, HV, CD, SW, MS, PV, JAH, JW, PM and AF provided a critical revision of the manuscript for important intellectual content. PM performed the statistical analysis as independent trial statistician. In addition, JPD contributed to the statistical analysis. JPD, AF and NH obtained funding. VH, SM, CLL, VK, MKLW, EJK, KS, NH, AM, ES, JP, CMK and SW provided administrative, technical, and material support. JPD, OWS, HV, PV, JW, PM and AF supervised aspects of the study. All authors read and approved the final version of the manuscript.

## Data sharing statement

Electronic recording, processing, and storage of the data were approved by the data protection officer of the Charité—Universitätsmedizin Berlin (data protection votes from May 28th 2008 and May 5th 2014). The raw datasets analysed during the current study are not publicly available because the patient's informed consent only permits the data analysis and publication by the investigators.

## Declaration of interests

The authors declare that they have no competing interest.
