# Supplementary material for: Blood–brain barrier opening as a predictor of epilepsy and mortality after subarachnoid haemorrhage
Source: eBioMedicine. 2025 Nov 11;122:106018. doi: 10.1016/j.ebiom.2025.106018 (PMC12651435; doi:10.1016/j.ebiom.2025.106018)
Supplement: Supplementary Figures [file mmc1.pdf]

SUPPLEMENTARY MATERIAL

Supplement to: Dreier et al. Blood-brain barrier opening as a predictor of epilepsy and mortality after subarachnoid haemorrhage

Table of contents:

Supplementary Figure 1 **2**

Supplementary Figure 2 **3**

Supplementary Figure 3 **5**

Supplementary Figure 4 **6**

Supplementary Figure 1

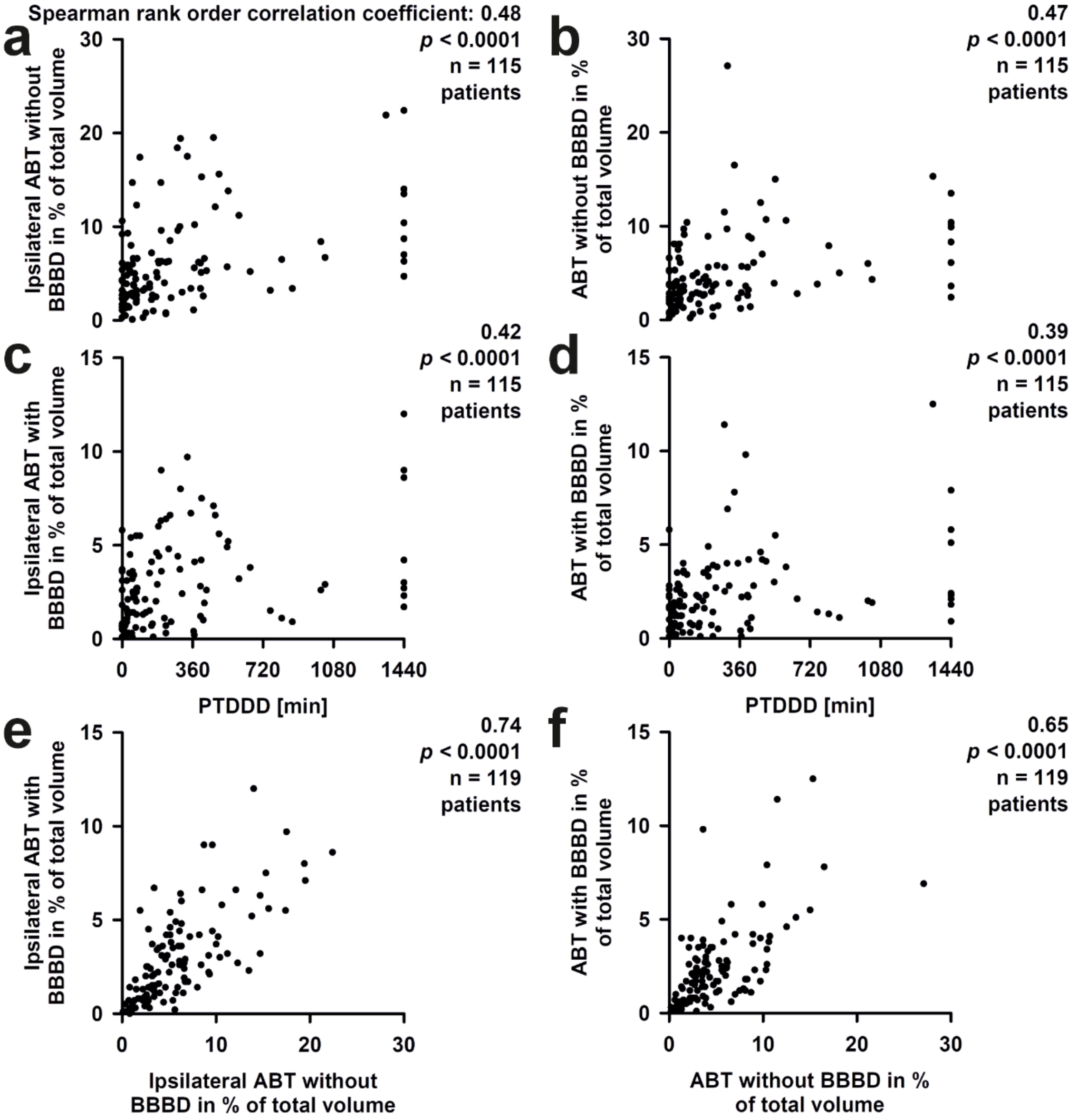

Supplementary Figure 1: Correlations between the two components of abnormal brain tissue (ABT) in the MRI at the end of neuromonitoring and peak total spreading depolarisation (SD)-induced depression duration of a recording day (PTDDD). Both semi-automatically segmented ipsilateral (a) and bilateral (b) ABT without blood-brain barrier dysfunction (BBBD) as a percentage of the ipsilateral or total intracranial volume correlated strongly with PTDDD. Ipsilateral (c) and bilateral (d) ABT with BBBD in the same MRI also correlated with PTDDD, but the correlations were somewhat weaker. The strongest correlations were found between the two MRI variables (e and f). The x-axes in (c) and (d) also apply to (a) and (b).

Supplementary Figure 2

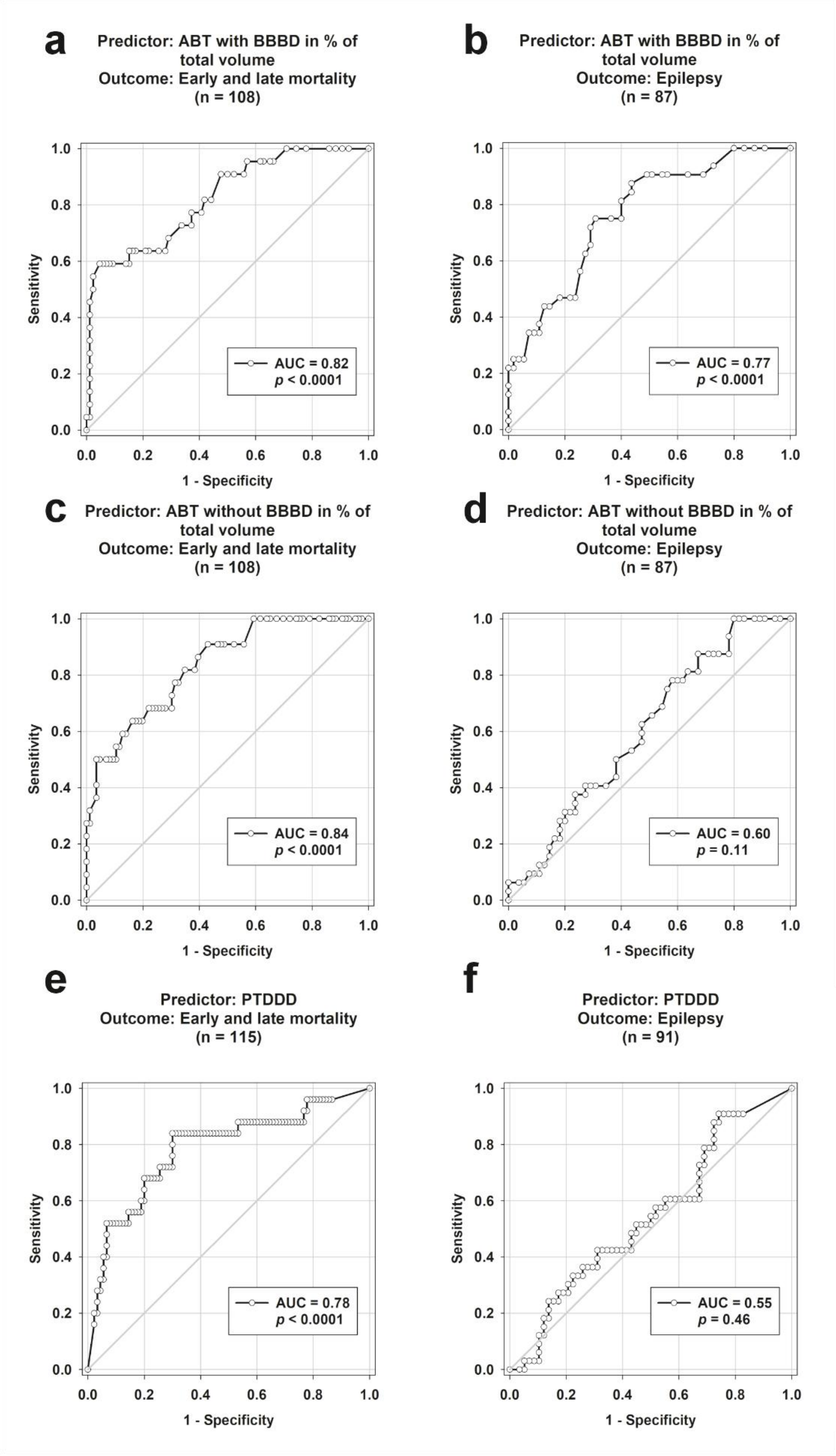

Supplementary Figure 2: Only abnormal brain tissue (ABT) with blood-brain barrier dysfunction (BBBD) as a percentage of intracranial volume in the MRI at the end of neuromonitoring is an indicator of both (1) combined early and late mortality after the neuromonitoring period and (2) late epilepsy. (a) Area under the receiver operating characteristic curve (AUC) for ABT with BBBD in the MRI at the end of neuromonitoring to indicate the combined early and late mortality after the neuromonitoring period (0·824, 95% confidence interval (95%CI): 0·724-0·923). (b) AUC for ABT with BBBD in the MRI at the end of neuromonitoring to indicate epilepsy (0·766, 95%CI: 0·665-0·867). (c) AUC for ABT without BBBD in the MRI at the end of neuromonitoring to indicate the combined early and late mortality after the neuromonitoring period (0·835, 95%CI: 0·746-0·925). (d) AUC for ABT without BBBD in the MRI at the end of neuromonitoring to indicate epilepsy (0·604, 95%CI: 0·485-0·723). (e) AUC for the peak total spreading depolarisation (SD)-induced depression duration of a recording day (PTDDD) to indicate the combined early and late mortality after the neuromonitoring period (0·784, 95%CI: 0·674-0·895). (f) AUC for PTDDD to indicate epilepsy (0·546, 95%CI: 0·424-0·669).

Supplementary Figure 3

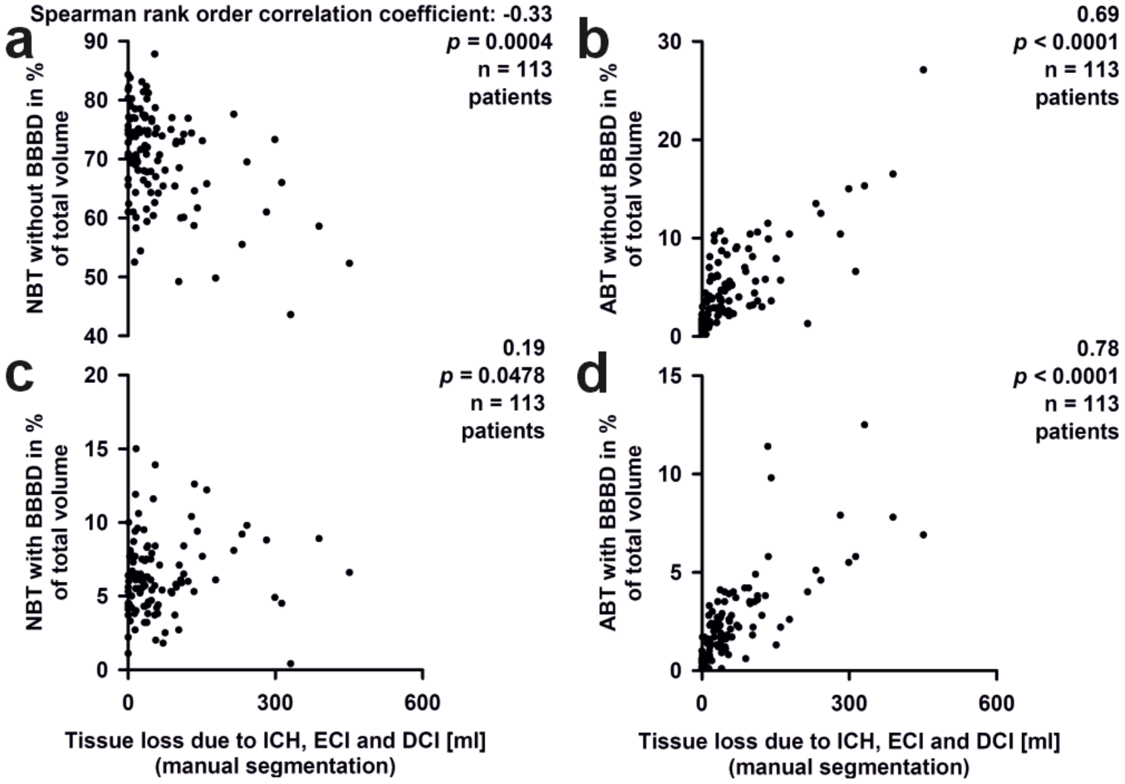

Supplementary Figure 3: Correlations between manually segmented tissue loss due to intracerebral hemorrhage (ICH), early cerebral ischaemia (ECI) and delayed cerebral ischaemia (DCI) and the semi-automatically segmented MRI variables at the end of monitoring. The volumes of the parenchymal lesions derived from manual segmentation were adopted from DISCHARGE-1 (24). Manually segmented tissue loss strongly correlated with $ABT_{BBBD}\%_{post\text{-}monitoring}$ (d). ABT, abnormal brain tissue; BBBD, blood-brain barrier dysfunction; NBT, normal brain tissue. The x-axes in (c) and (d) also apply to (a) and (b).

Supplementary Figure 4

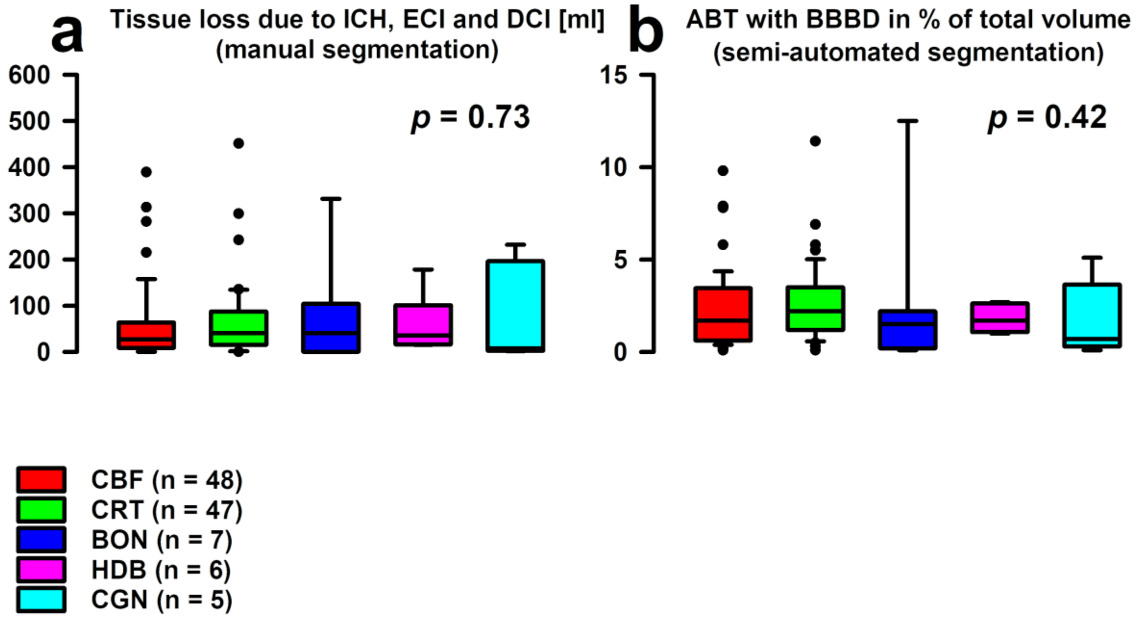

Supplementary Figure 4: Comparison of the different study centres with regard to manually and semi-automatically segmented neuroimaging variables at the end of monitoring. Comparisons were done using Kruskal-Wallis One Way Analysis of Variance on Ranks. For the comparison between the different study centres in (a), the volumes of the parenchymal lesions due to intracerebral haemorrhage (ICH), early cerebral ischaemia (ECI) and delayed cerebral ischaemia (DCI) derived from manual segmentation were adopted from DISCHARGE-1 (24). All patients were compared for whom both the manually segmented variable and the semi-automatically segmented variable were available. The different MRI scanners may have affected the results. However, the comparison between the different study centres does not indicate that this influence was substantial. Study centres: CBF, Campus Benjamin Franklin, Charité-Universitätsmedizin Berlin; CRT, Campus Virchow Klinikum, Charité-Universitätsmedizin Berlin; BON, University of Bonn; HDB, University Hospital Heidelberg; CGN, University of Cologne. ABT, abnormal brain tissue; BBBD, blood-brain barrier dysfunction; DCI, delayed cerebral ischaemia; ECI, early cerebral ischaemia; ICH, intracerebral haemorrhage; NBT, normal brain tissue.
